# Single-cell and spatial transcriptomics reveal alterations in trophoblasts at invasion sites and disturbed myometrial immune microenvironment in placenta accreta spectrum disorders

**DOI:** 10.1186/s40364-024-00598-6

**Published:** 2024-06-03

**Authors:** Kaiyuan Ji, Yunshan Chen, Xiuyu Pan, Lina Chen, Xiaodi Wang, Bolun Wen, Junjie Bao, Junmin Zhong, Zi Lv, Zheng Zheng, Huishu Liu

**Affiliations:** 1 Guangzhou Key Laboratory of Maternal-Fetal Medicine, Guangzhou Women and Children’s Medical Center, Guangzhou Medical University, No. 9 Jinsui Road, Guangzhou, China; 2 Institute of Reproductive Health and Perinatology, Guangzhou Women and Children’s Medical Center, Guangzhou Medical University, Guangzhou, China

**Keywords:** Placenta accreta spectrum, Spatial transcriptomes, Single-cell sequence, Trophoblast, Myometrium

## Abstract

**Background:**

Placenta accreta spectrum disorders (PAS) are a severe complication characterized by abnormal trophoblast invasion into the myometrium. The underlying mechanisms of PAS involve a complex interplay of various cell types and molecular pathways. Despite its significance, both the characteristics and intricate mechanisms of this condition remain poorly understood.

**Methods:**

Spatial transcriptomics (ST) and single-cell RNA sequencing (scRNA-seq), were performed on the tissue samples from four PAS patients, including invasive tissues (ST, *n* = 3; scRNA-seq, *n* = 4), non-invasive normal placenta samples (ST, *n* = 1; scRNA-seq, *n* = 2). Three healthy term pregnant women provided normal myometrium samples (ST, *n* = 1; scRNA-seq, *n* = 2). ST analysis characterized the spatial expression landscape, and scRNA-seq was used to identify specific cellular components in PAS. Immunofluorescence staining was conducted to validate the findings.

**Results:**

ST slices distinctly showed the myometrium in PAS was invaded by three subpopulations of trophoblast cells, extravillous trophoblast cells, cytotrophoblasts, and syncytiotrophoblasts, especially extravillous trophoblast cells. The pathways enriched by genes in trophoblasts, smooth muscle cells (SMC), and immune cells of PAS were mainly associated with immune and inflammation. We identified elevated expression of the angiogenesis-stimulating gene PTK2, alongside the cell proliferation-enhancing gene EGFR, within the trophoblasts of PAS group. Trophoblasts mainly contributed the enhancement of HLA-G and EBI3 signaling, which is crucial in establishing immune escape. Meanwhile, SMC regions in PAS exhibited upregulation of immunomodulatory markers such as CD274, HAVCR2, and IDO1, with CD274 expression experimentally verified to be increased in the invasive SMC areas of the PAS group.

**Conclusions:**

This study provided information of cellular composition and spatial organization in PAS at single-cell and spatial level. The dysregulated expression of genes in PAS revealed a complex interplay between enhanced immune escape in trophoblasts and immune tolerance in SMCs during invasion in PAS. These findings will enhance our understanding of PAS pathogenesis for developing potential therapeutic strategies.

**Supplementary Information:**

The online version contains supplementary material available at 10.1186/s40364-024-00598-6.

## Background

PAS is defined as an abnormal trophoblast invasion into the myometrium, and even to or beyond the serosa [1–3]. Multiple morbidities were associated with PAS including intractable postpartum hemorrhage, massive blood transfusion, and even maternal death, with more possibilities of hysterectomy, iatrogenic surgical injury to bowel or bladder [4]. Epidemiological research on PAS identifies a history of prior caesarean delivery as the predominant risk factor for the development of PAS [5–7]. The leading hypothesis suggests that previous uterine surgery affecting the endometrial-myometrial interface results in defective decidualization at the scar site [8, 9]. This abnormality allows the placental anchoring villi to improperly attach to the myometrium, leading to increased trophoblast invasion [10, 11]. PAS cases are also found in primigravid women who have had no previous uterine surgeries [12]. It’s speculated that these rare cases might be due to uterine conditions like adenomyosis, submucous fibroids, or bicornuate uterus, leading to microscopic endometrial defects that disrupt normal endometrial function and result in abnormal placental attachment [13]. The loss of decidua, increased trophoblast invasiveness, and abnormal remodeling of uterine spiral arteries are viewed as the three key pathophysiological factors contributing to PAS. These elements interact and influence each other, leading to the condition’s development [14, 15].

Decidua protects the embryo from from being attacked by maternal immune cells. Further, the decidua has to allow a very controlled invasion of the trophoblast. Incomplete decidua in the invasive area results in direct contact between trophoblasts and myometrium, facilitating the interaction of immune environments from both sides and forming a distinctive microenvironment [16, 17]. The microenvironment of invasive parts in PAS comprises a diverse array of cell populations, including trophoblasts, stromal cells, immune cells, and vascular components [18–20]. The hallmark of these invasive parts is characterized by sophisticated and multifaceted intercellular interactions. These interactions are governed by a network of signaling pathways, mediated by cytokines, growth factors, and immune modulators, along with critical elements of the extracellular matrix. These constituents collectively contribute to the intricate and evolving nature of the microenvironment in PAS, influencing pathological progression and cellular behavior within these invasive regions [21, 22].

Initial investigations into PAS were largely conducted through techniques such as Western Blotting, Immunohistochemistry, and quantitative PCR to analyze relevant tissues. While these methodologies facilitated the identification of specific features associated with PAS, their limited throughput restricted our ability to gain a comprehensive understanding, offering only a fragmentary glimpse into the complex biology of this process. The high throughput transcriptional signature of PAS has not been reported until recent years (including mRNAs, miRNAs, lncRNAs, and circRNAs), probably as a consequence of lacking specimens from both confirmed PAS patients and appropriate controls [23–26]. Previous reports on PAS-related bulk transcriptomic and proteomic analyses revealed that the differentially expressed genes were mainly related to cell proliferation, inflammation, migration and vascular development; Quantitative PCR and immunohistochemical staining confirmed down-regulated WNT5A and MAPK13 in PAS [23, 24]. scRNA-seq analysis was conducted on both the representative invasive parts and the normal part obtained from the same placenta affected by PAS [19]. This comprehensive analysis provided valuable insights into the pathological landscape of invasive PAS placenta. GeoMX Digital Spatial Profiler were used to resolved spatial transcriptome in PAS, but this technique involves manually selecting areas, which introduces selection bias and does not provide comprehensive transcriptomic information across the entire tissue [20]. Additionally, this work does not explore the role of immunity, which plays a central role in the implantation process. There has yet to be a study examining the alterations in the myometrial immune environment following trophoblast invasion in PAS. The lack of normal myometrium samples as controls has hindered direct comparisons of changes at the single-cell level within the myometrial region.

Advanced high-resolution molecular detection techniques, such as spatial transcriptomics (ST) and scRNA-seq, are the powerful tools to investigate the mechanism and microenvironment involved in PAS [27–30]. These cutting-edge techniques enable researchers to explore the spatial distribution of gene expression patterns within the placenta and evaluate the heterogeneity of cell populations at a single-cell level. Here, we aimed to explore the landscape and specific alterations within the maternal–fetal interface microenvironment in PAS using unbiased 10X scRNA-seq and 10X Visium ST. This approach provides a comprehensive overview of PAS, revealing its spatial heterogeneity in detail. We also used placental tissues from non-invasion areas and normal myometrium in term pregnancy as controls. This allows us to investigate how these cell populations collaboratively influence tissue phenotypes, potentially laying the groundwork for future research on biomarkers and therapies for PAS.

## Methods

### Sample collection

The PAS cases were prenatally diagnosed as placenta previa combined with increta or percreta using ultrasound and MRI scans [31]. All cases were PAS level II classification, which was also confirmed by the observations in surgery and the pathology after surgery. The parts of placenta invading the myometrium in PAS were obtained from the removed invaded placenta and myometrial mixed tissue, where uncontrolled bleeding occurred [32, 33]. Normal placenta samples were collected from the detached placenta at the non invaded position in patients with PAS. The normal myometrium tissues were obtained from women who underwent uncomplicated cesarean deliveries at term non-labour (≥ 37 weeks of gestation) and did not have any pregnancy-associated complications [34, 35].

Placental samples were obtained from four PAS patients, including PAS-invaded region (PAS group) and non-invaded sites (placenta group). In the PAS group, three samples were used for ST (the ST failed for the fourth patient)and four samples for scRNA-seq. In the placenta group, one sample was used for ST and two samples for scRNA-seq. Additionally, normal myometrium samples were obtained from three term non-labor women (myometrium group: ST, *n* = 1; scRNA-seq, *n* = 2), these samples were also used in our previous publications [35, 36]. The sample collection procedure adhered to the previously described protocol [35, 36]. The clinical information for the PAS samples was described in Table S1. The tissues were immersed in physiologic saline and transported to the laboratory for scRNA-seq, ST, hematoxylin and eosin staining (H&E), and immunofluorescence. The study was conducted in accordance with the ethical guidelines and was reviewed and approved by the Ethics Committee of Guangzhou Women and Children Medical Center (approval number: 202012800). Written informed consent was obtained from all patients/participants prior to their involvement in the study.

### scRNA-seq and ST sequencing

The cell isolation and construction of the scRNA-seq library were performed as previously described [35]. Sequencing was carried out on an Illumina NovaSeq 6000 (Annoroad Gene Technology, Beijing, China) using paired-end multiplexing with a read length of 150bp.

For ST, fresh samples were rinsed twice in DMEM (Gibco) and regions of interest, measuring no more than 6 mm × 6 mm, were excised. These excised tissues were embedded in OCT (optimal cutting temperature) and stored at -80℃. Then tissues were sectioned to a thickness of 10 μm at -15℃ using a Leica cryostat. These sections were mounted on highly adhesive slides for sample selection, and confirmed for optimal positioning through H&E staining. Subsequently, the tissues sections were fixed onto 10X Visium Tissue Optimization Slides or Visium Spatial Gene Expression Slides (10X Genomics), followed by cell lysis and library preparation for sequencing as previously described [35, 36]. The integrity and quality of the library DNA fragments were evaluated using an Agilent 2100 instrument. The libraries were sequenced using an Illumina Novaseq 6000 (Annoroad Gene Technology, Beijing, China) with a minimum sequencing depth of 100,000 reads per spot and paired-end 150 bp sequencing.

### scRNA-seq analysis

The sequencing data obtained were converted into the FASTQ format using the bcl2fastq software (v2.20). The CellRanger pipeline was used for sample demultiplexing, barcode processing, and gene counting (https://support.10xgenomics.com/). The sequencing data were aligned to the human reference genome (Ensembl genome GRCh38). The resulting output from Cell Ranger was imported into Seurat (v4.3.0) for dimensional reduction and subsequent analysis [37]. To exclude low-quality cells, we applied filters to remove cells with either fewer than 2,000 or more than 30,000 UMIs, as well as cells with over 6,000 gene features. Additionally, any cells exhibiting more than 20 percent mitochondrial genes were also excluded. We also identified doublet cells, which constituted 7.6% of the total, using DoubletFinder [38]. Only singlet cells were included in the subsequent analysis. We calculated the anchors of different datasets using the “FindIntegrationAnchors” function. “Anchor.features” was set to 3,000, “k.anchor” was set to 20, the “IntegrateData” function was used to integrate the individual datasets and the top 30 dimensions were used for the anchor weighting procedure. The nearest neighbors were constructed using the “FindNeighbors” function and the top 20 dimensions of reduction were used as input. For subpopulation of celltypes, “Harmony” were used for integration of each groups [39]. Then, the clusters of cells were identified with resolution 1 (all cell types) or 0.3 (subpopulation) and the cluster markers were calculated using the “FindAllMarkers” function, and the cell types were assigned based on their specific gene expression. Pathway enrichment analyses were performed using Gene Set Enrichment Analysis (GSEA) with the WikiPathways databases [40].

### Spatial transcriptome analysis

The histology images and FASTQ files were processed using the Space Ranger pipeline (https://support.10xgenomics.com/), and the sequence data were aligned to the human reference genome (Ensembl genome GRCh38). The resulting output was then imported into Seurat for further analysis. The spatial transcriptome data were loaded using the “Load10X_Spatial” function and we performed the NormalizeData, FindVariableFeatures, ScaleData workflow using the “SCTransform” function, as well as the dimensionality reduction, clustering and visualization by Seurat. We integrated the scRNA-seq with the ST and predicted the cell types using the “FindTransferAnchors” and “TransferData” function. Then, we defined the maximum ratio of predicted cell types as spot types. To characterize the enrichment scores of pathways at spatial level, the function “AddModuleScore” of Seurat was used to calculate the enrichment scores and the enrichment results was visualized by “SpatialFeaturePlot”.

### Cell–cell communication analysis

The expression matrices of scRNA-seq data were processed using the Seurat package. Cell–cell communication networks were identified and visualized using the CellChat (V1.6.1) package, following the standard workflow [41]. The CellChatDB, a molecule interaction database, was used for the analysis.

### Immunofluorescence staining

Immunofluorescence and multiplex immunofluorescence staining were conducted following previously described protocols [35, 42]. We used the following primary antibodies: α-SMA/ACTA2 (1:300; ab7817, abcam), CK-7/KRT7 (1:200; ab154334, abcam), PD-L1/CD274 (1:2500; PA5-20343, INVTROGEN), INO1(1:2000; ab211017, abcam), TIM-3/HAVCR2 (1:1000; ab241332, abcam), EBI3 (1:5000; NBP-76976, Novus Biologicals), HLA-G (1:7500; ab283278, abcam), and WNT4 (1:500; GTX101085, GeneTex). Alexa Fluor 647 goat anti-mouse secondary antibody (1:500; ab150115, abcam) and Alexa Fluor 488-IgG goat anti-rabbit secondary antibody (1:500; ab150077, abcam) were used for detection. A goat anti-mouse/rabbit multiplex IHC detection kit (ZENBIO) was used for multiplex immunofluorescence staining with goat anti-mouse/rabbit HRP polymer as the secondary antibody. Finally, the sections were counterstained with the nuclear dye DAPI and visualized using a Leica DMi8 fluorescence microscope.

### Statistical analysis

All statistical analyses and figure creation were performed using R (v4.1.1). The differential expressed genes (DEGs) among groups were analyzed using “MAST” function in the Seurat package [43]. An adjusted *p*-value (*p*_val_adj) < 0.05 was considered statistically significant. Statistical differences between groups were assessed using a t test with two-sided in Immunofluorescence staining. *P*-value < 0.05 was statistically significant.

## Results

### Cellular composition and gene expression localization in PAS tissues revealed by scRNA-seq and ST

**Fig. 1:**
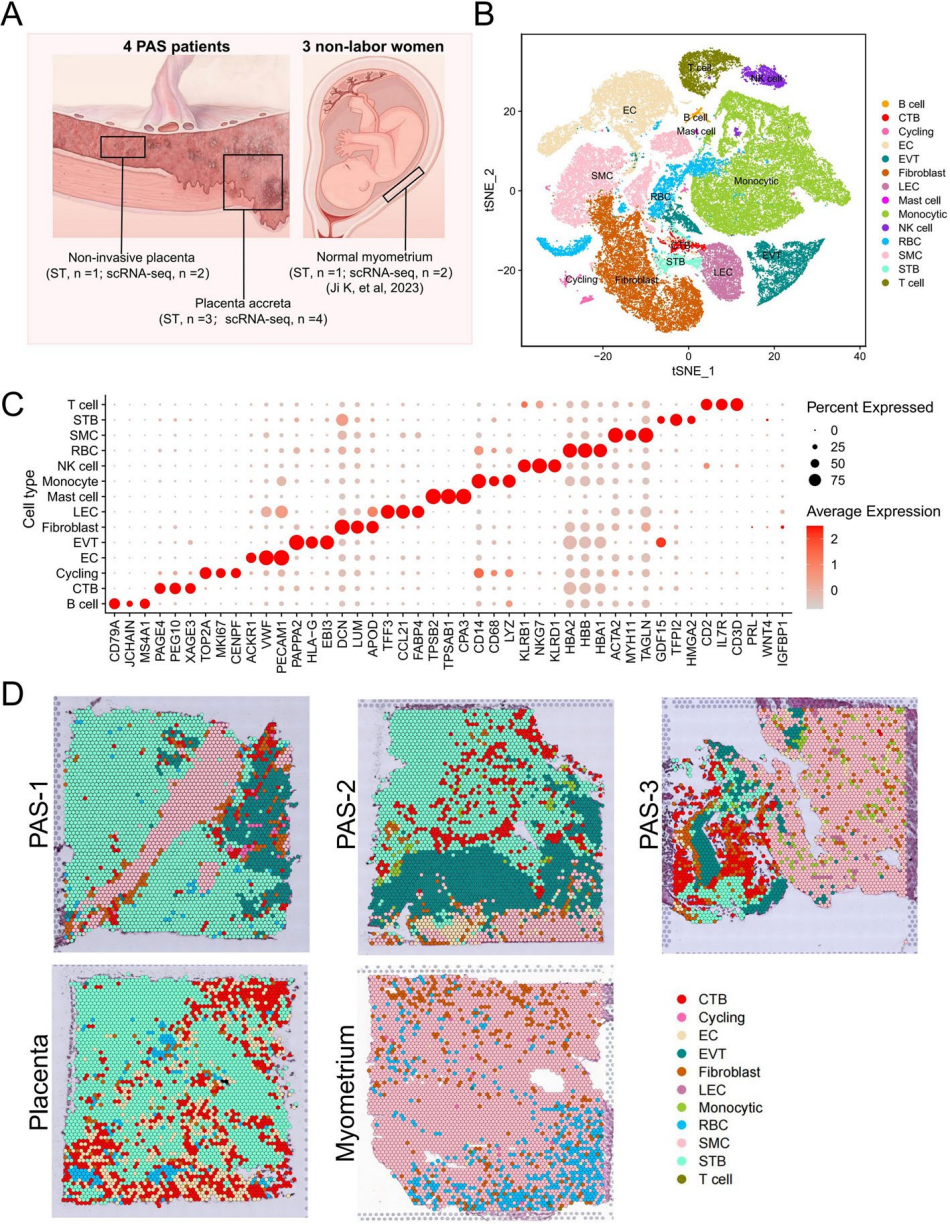
Overall landscapes of the scRNA-seq and ST in invasive PAS basal plate, normal placenta and myometrium. **A** The experimental design involved obtaining representative parts for ST and scRNA-seq. Placental samples were obtained from 4 PAS patients, including PAS-invaded region (PAS group: ST, *n* = 3; scRNA-seq, *n* = 4) and non-invaded sites (placenta group: ST, *n* = 1; scRNA-seq, *n* = 2). Normal myometrium samples were obtained from 3 term non-labor women (myometrium group: ST, *n* = 1; scRNA-seq, *n* = 2). **B** tSNE plot of the major cell populations in the scRNA-seq of PAS, normal placenta and myometrium groups. CTB, cytotrophoblast; EC, endothelial cell; EVT, extravillous trophoblast; LEC, lymphatic endothelial cell; RBC, red blood cell; SMC, smooth muscle cell; STB, syncytiotrophoblast. **C** Dot plot depicts the expression of canonical markers for major cell populations in the scRNA-seq data. The color of each dot indicates the expression level of the marker gene, and the size of the dot reflects the percentage of cells expressing the marker genes across various cell types. **D** Spatial feature plots of the cell types’ prediction in the invasive parts of PAS, non-invasive placenta and normal myometrium

We choose four patients who experienced PAS at the site of scarring due to a previous cesarean section. These cases were confirmed as level II PAS through ultrasound and MRI (magnetic resonance imaging), as well as intraoperative observations (accreta lesion clearance, Nausica suture, uterine repair surgery) and subsequent pathological validation (Figure S1A). We selected the invasive tissues containing trophoblasts and myometrium from these four patients, along with placenta from non-invasive areas at the same level as controls. This was to study the differences in trophoblasts between the invasive and non-invasive areas. Due to ethical requirements and the protection of pregnant women, we were unable to collect corresponding non-invasive myometrial areas from these four patients. Therefore, we used normal myometrium from term pregnancy from our previous studies as a control for comparison [35], to investigate changes in the myometrial stromal cells and immune environment following excessive invasion by trophoblasts. Our samples are divided into three groups: the invasive parts of PAS (PAS group), non-invasive placenta of PAS (placenta group) and normal myometrium (myometrium group) (Fig. 1A and S2A). The cells of scRNA-seq were clustered using t-distributed stochastic neighbor embedding (t-SNE) (Fig. 1B), and the major cell types were identified with the expression of canonical marker genes [19, 35, 44, 45], including three types of trophoblasts: EVT (HLA-G, PAPPA2) [19, 45–48], STB (GDF15, TFPI) [46, 49], CTB (PAGE4) [46, 50]; stromal cells: SMC (ACTA2) [35], fibroblast (DCN) [35], endothelial cell (EC) (VWF) [35], lymphatic endothelial cell (LEC) (TFF3) [35]; immune cells: T cell (CD3D) [19, 35], B cell (CD79A) [19, 35], NK cell (KLRB1) [51], monocyte (CD14) [35, 52], mast cell (TPSB2) [35]; cells in cell cycle (cycling) (TOP2A) [53] and red blood cell (RBC) (HBA2) [54] (Fig. 1C and S2B). A small number of cells expressed the decidua markers (PRL and WNT4) [55]; therefore, we performed WNT4 immunofluorescence to indicate the deficiency of decidua in the invasive parts of PAS (Figure S2C). We integrated the scRNA-seq and ST data to calculate the cell type prediction probabilities of each spot using factor analysis via Seurat [56] (Figure S3A). The cell type that registers the highest score at each spot is designated as the cell type for that spot. Our results revealed a close association between SMCs and three types of trophoblasts in the three invasive tissue samples (Fig. 1D). The major cell types in the placenta were STB, CTB, and EC, and those in myometrium were SMCs and fibroblasts. In addition, we isolated cycling cells and conducted cell type identification. The results show that the predominant cell types of cycling cells are monocytic and T cells. Notably, there are more cycling T cells in PAS than in the placenta (Figure S4A).

### Cell heterogeneity and DEGs (differentially expressed genes) between PAS and placenta or myometrium

**Fig. 2:**
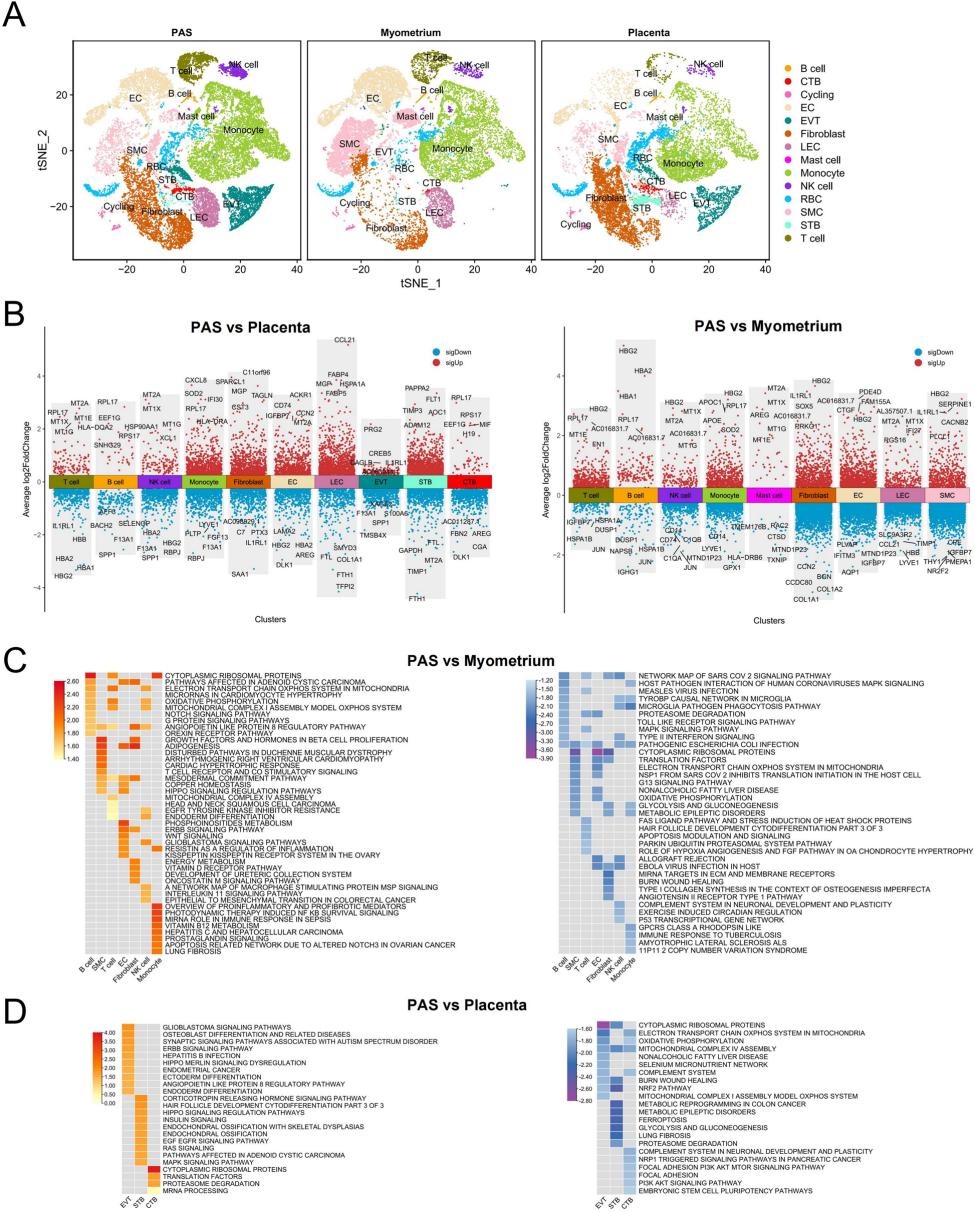
Differences in cellular composition and gene expression among the PAS and normal placenta, and myometrium groups. **A** t-SNE plot of the major cell populations in the scRNA-seq data segregated by groups. Invasive parts of PAS (PAS), non-invasive placenta (placenta) and normal myometrium (myometrium). **B** Volcano plots of the differentially expressed genes and shows the top five upregulated and downregulated genes in major cell populations. Red represents upregulated, and blue represent down-regulated in the PAS group. Log_2_FoldChange cutoff shown in figures is 0.25. **C** Heatmap reveals the NES (Normalized Enrichment Score) of top10 up (red) and down (blue) significantly (FDR < 0.05) changed pathways in SMCs, fibroblasts, EC and major immune cells between PAS and myometrium groups. **D** Heatmap reveals the NES (Normalized Enrichment Score) of top10 up (red) and down (blue) significantly (FDR < 0.05) changed pathways in three types of trophoblasts between PAS and placenta groups

We split the t-SNE plot of scRNA-seq data into PAS, placenta, and myometrium groups to determine the difference between PAS and corresponding normal tissue (Fig. 2A). For trophoblasts, invasive parts of PAS exhibited a higher abundance of EVTs and a lower abundance of STBs compared to the placenta group, which aligned with our current understanding of trophoblasts in PAS. Further, we separately calculated the DEGs between the PAS and placenta groups, and between the PAS and myometrium groups (Fig. 2B; Table S2). To better display the changing functions and pathways, we performed GSEA to compare the occurrence of stromal cells (SMC, fibroblast and EC) and immune cells between the PAS and myometrium groups, as well as the three trophoblast types between the PAS and placenta groups (Fig. 2C and D). In the PAS cohort, we observed significant upregulation of the ERBB and WNT signaling pathways in EC. Conversely, T cells, NK cells, and monocytes exhibited downregulation in pathways related to infection, allograft rejection, and immune responses. Additionally, in EVT within the PAS group, ERBB and angiopoietin pathways were prominently upregulated. However, aerobic respiration processes such as oxidative phosphorylation and the electron transport chain were suppressed in PAS EVT. In STB, the EGFR and RAS signaling pathways were upregulated, whereas processes associated with ferroptosis, glycolysis and gluconeogenesis were downregulated. Furthermore, in CTB within the PAS group, focal adhesion and the PI3K-AKT pathway showed downregulation, highlighting distinct regulatory alterations in different trophoblast subtypes associated with PAS.

### Characteristics and differences of cellular communication in PAS

**Fig. 3:**
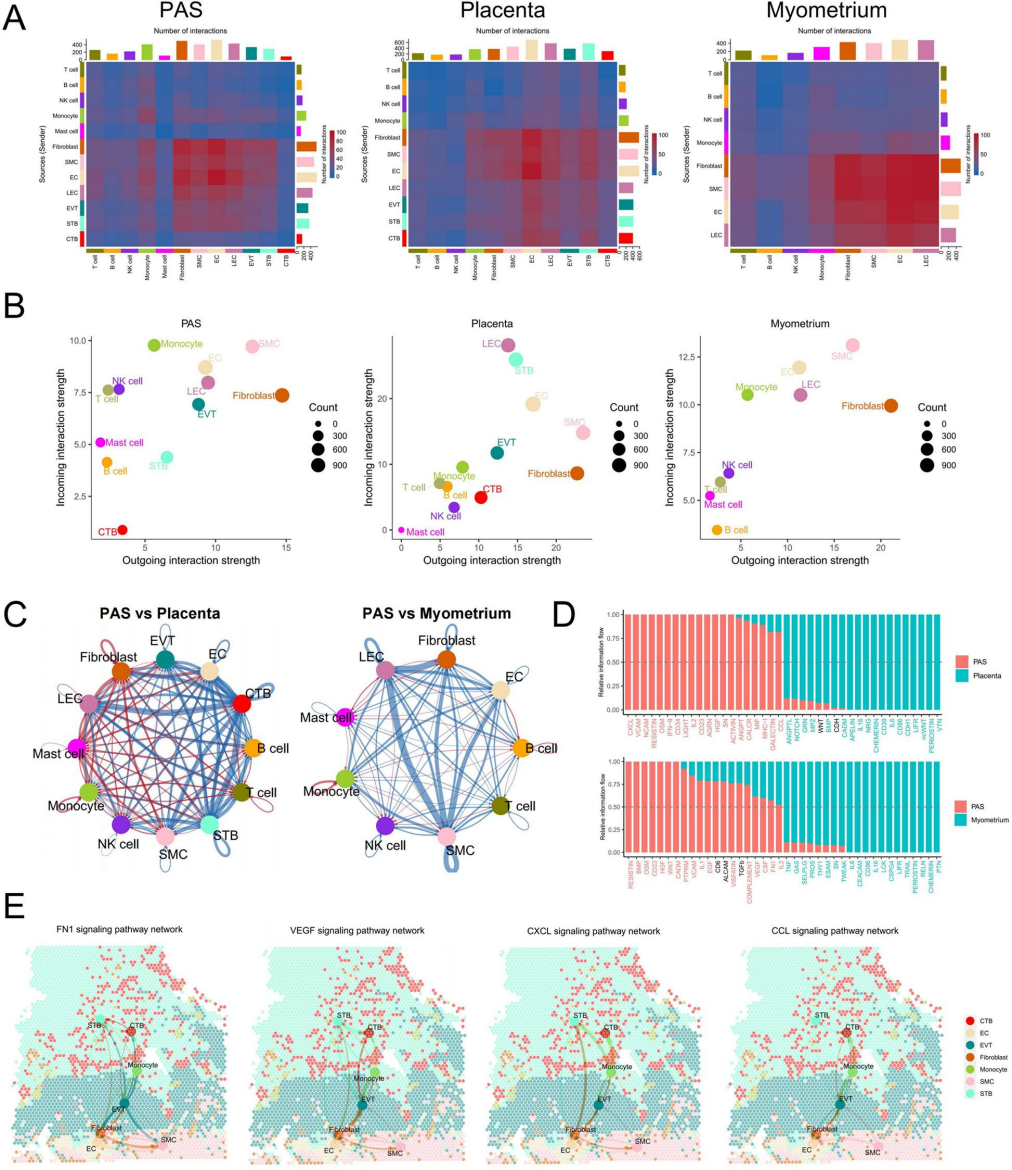
Cell–cell communication interactions in PAS. **A** Interaction numbers among each cell type in the PAS, placenta and myometrium groups. **B** Scatter plots of the interaction strength of outgoing and incoming signals for each cell population in the PAS, placenta and myometrium groups. **C** Circle plot illustrates the differential number of interactions between any two cell types in the PAS group compared with the placenta or myometrium groups. The edges connecting the cell types are color-coded, with red indicating increased signaling in the PAS group compared with the placenta or myometrium groups, and blue indicates the opposite trend. The thickness of the lines represents the number of interactions. **D** Information flow of each of the top 20 signaling pathways in the PAS group compared with the placenta or myometrium groups. Red indicates significantly (*p* < 0.05) increased signaling in the PAS group compared with the placenta or myometrium groups, blue indicates the significantly (*p* < 0.05) opposite trend, and black indicates no significantly difference (*p* > 0.05). **E** Spatial visualization of the FN1, VEGF, CXCL, and CCL pathways of cell–cell communication in the ST data of the PAS-2 sample. The thickness of the lines represents the number of interactions

When trophoblasts abnormally invade the myometrium, they come into contact and proximity with SMCs and immune cells, leading to unique intercellular communication. To investigate the cellular signaling involved in this, we conducted an analysis of their communication using our scRNA-seq data using CellChat R package. These results revealed the differential cell–cell interactions in various cell types among the different groups. In the PAS group, cell types including EC, EVT, fibroblasts, LEC, monocytes, SMC, and STB were more actively involved in cellular interactions compared to other cell types within the same group. Specifically, EC, EVT, fibroblasts, LEC, and STB played prominent roles in these interactions, more so than in other cell types. Within the normal myometrium group, EC, monocytes, fibroblasts, and SMC were the most active contributors to cellular interactions compared to their counterparts (Fig. 3A). Next, we analyzed the incoming and outgoing interaction strength of all cell types in the three groups. SMC showed strong cell–cell interaction intensity in all three groups (Fig. 3B), indicating its important role in cell communication. We calculated the differential number of interactions in the cell–cell communication network between the PAS and placenta groups and PAS and myometrium groups (Fig. 3C). The interactions between trophoblasts and immune cells were lower in invasive parts of PAS than those in the placenta group. The interaction between SMCs with other cells (except monocytes and mast cells) was lower in the PAS group than in the myometrium group. The top 20 significantly different pathways between the PAS and placenta groups and PAS and myometrium groups revealed that inflammation-related pathways exhibit both upregulation and downregulation in the PAS group (Fig. 3D and S4B). In PAS, the chemerin, IL6, TNF and IL16 pathways exhibited a significant decrease, whereas the MHC-I, OSM and IL2 pathways were increased. This suggests that a substantial number of inflammation-related genes have undergone changes, seemingly establishing a new equilibrium between anti-inflammatory and pro-inflammatory factors. The HGF (Hepatocyte Growth Factor), VEGF (Vascular Endothelial Growth Factor), and EGF (Epidermal Growth Factor) pathways were up-regulated in PAS, which primarily involved in cell growth, cell proliferation, angiogenesis and survival [57, 58]. The TGF-β pathway, known for its involvement in invasion and immunosuppression [59–61], showed no difference in PAS compared to the myometrium. We spatially displayed some significantly crucial cell communication in the ST data (Fig. 3E). In PAS, cell communication related to FN1 pathway predominantly involves EVTs, SMCs, and Fibroblasts, with the pathway primarily influencing proliferation, migration, and invasion. VEGF-related cell communication is largely centered around EVTs. Within the CCL and CXCL pathways, EVTs, CTBs, fibroblasts, and SMCs are identified as major contributors in the invasive components of PAS. The interactions between three types of trophoblasts and various immune and fibroblast cells are significantly enhanced. We have displayed the upregulated intercellular interactions in the PAS group, involving ligand-receptor pairs such as FB1-CD44, COL4A1-CD44, and PGF-VEGFR1 (Figure S4C).

### Investigating the gene expression pattern of trophoblasts on angiogenesis and hypoxia in PAS

**Fig. 4:**
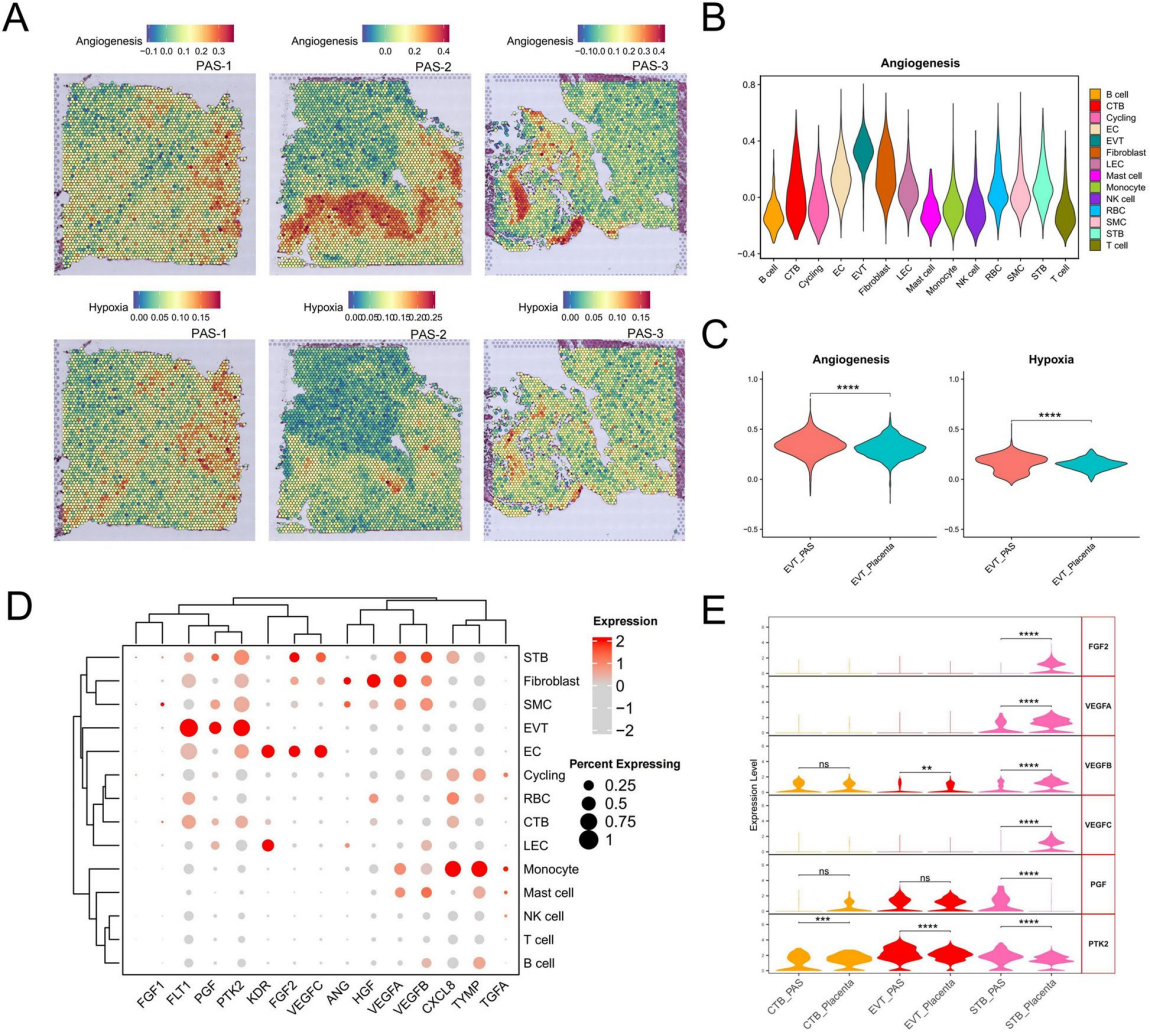
Analysis of angiogenesis and hypoxia related genes in trophoblasts. **A** Enrichment score of angiogenesis and hypoxia pathways of each spot in the ST data. Gradient from blue (low score) to red (high score). **B** Enrichment score of angiogenesis pathways of each cell type in the scRNA-seq data. **C** Violin plot of enrichment score of angiogenesis and hypoxia pathways in EVTs of PAS and placenta groups in the scRNA-seq data (****, *p* < 0.0001). **D** Dot plot depicts the expression of canonical angiogenesis related factors and receptors in each cell type, as revealed in the single-cell RNA sequencing (scRNA-seq) data. The color of each dot indicates the expression level of the marker gene, and the size of the dot reflects the percentage of cells expressing the marker genes across various cell types. **E** Violin plot of the expression of angiogenesis related factors in three types trophoblast (ns, *p* > 0.05; **, *p* < 0.01; ***, *p* < 0.001; ****, *p* < 0.0001). Genes not expressed in trophoblast cells are not shown

Increased vascularity and abnormal blood flow patterns are primary characteristics of PAS and currently serve as the main indicators used in the initial step of detection [13]. Referencing the cell types and their locations in Fig. 1D, we observed that both angiogenesis and the closely associated hypoxia pathways are enriched at spots where EVT are located (Fig. 4A). In the scRNA-seq data, EVT exhibited the highest enrichment scores (Fig. 4B). Compared to the placenta group, both angiogenesis and hypoxia pathways were upregulated in the PAS group (Fig. 4C). We demonstrated the expression of core angiogenesis factors and receptors across different cell types [62], noting that FGF is primarily expressed in STB and EC (Fig. 4D). HGF is mainly found in fibroblasts, while PGF is predominantly expressed in EVT. Additionally, EVT also express FLT1, and STB express factors of VEGF series. Analyzing factors expressed in more than 50% of cells, we found that, relative to the placenta, the VEGF series factors in STB tend to decrease in PAS, whereas PGF shows an increase (Fig. 4E). KDR, as a major receptor of angiogenesis, is primarily expressed in EC and LEC. In addition, we found PTK2 in EVT and STB was up-regulated in PAS group. PTK2, also known as Focal Adhesion Kinase (FAK), plays a significant role in angiogenesis, which is the formation of new blood vessels [63].

### Gene expression changes of invasion-related genes in trophoblast of PAS

**Fig. 5:**
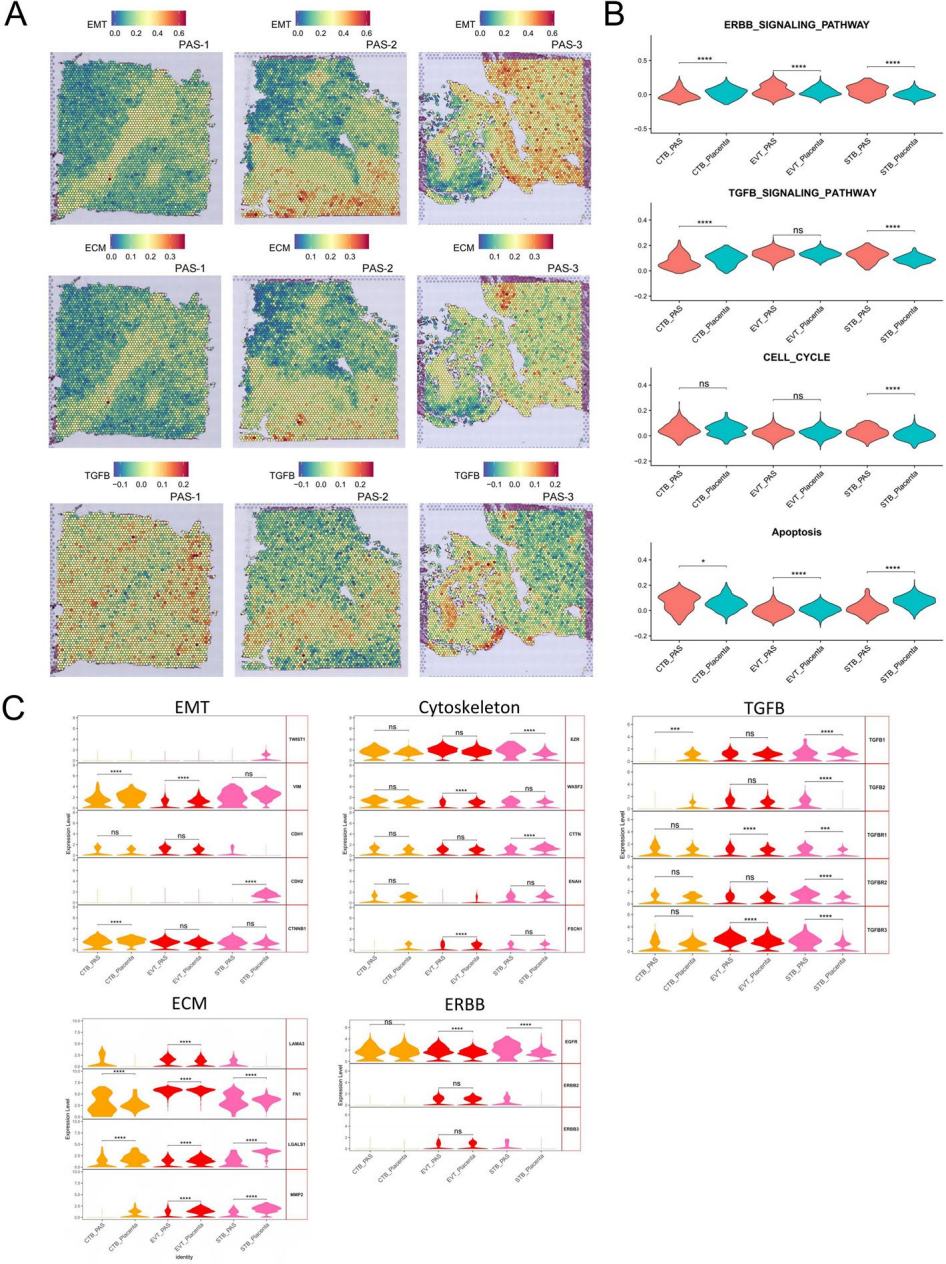
Analysis of pathways and genes associated with invasion in trophoblast. **A** Enrichment score of epithelial–mesenchymal transition (EMT), extracellular matrix (ECM) and TGFB pathways of each spot in the ST data. Gradient from blue (low score) to red (high score). **B** Violin plots of enrichment score of ERBB, TGFB, cell cycle and apoptosis pathways in EVTs of PAS and placenta groups in the scRNA-seq data (ns, *p* > 0.05; *, *p* < 0.05; ****, *p* < 0.0001). **C** Violin plots of the expression of the core genesof EMT, Cytoskeleton, TGFB, ECM and ERBB pathway in three types trophoblast (ns, *p* > 0.05; ***, *p* < 0.001; ****, *p* < 0.0001). Genes not expressed in trophoblast cells are not shown

Abnormal invasion of trophoblasts into the myometrium is a key characteristic of PAS [18]. However, there is still debate regarding whether the invasiveness of trophoblasts in PAS is increased, especially in cases where PAS is caused by damage to the myometrium or decidua [64]. We investigated the invasion-related pathways and the expression changes in the invasion-related genes in trophoblasts [65, 66]. Through the analysis of ST data, we found that epithelial-mesenchymal transition (EMT) and extracellular matrix (ECM) primarily occur at spots of EVT and SMC (Fig. 5A). As critical pathways in invasion, TGFB is mainly enriched at EVT sites. Compared to the placenta group, we observed a decrease in apoptosis and an increase in the ERBB pathway in EVT, with no significant change in the TGFB pathway (Fig. 5B). In STB, both the ERBB and TGFB pathways are upregulated in PAS, along with an up-regulation in the cell cycle and a decrease in apoptosis. We displayed the expression of genes of five core pathways associated with invasion (Fig. 5C). This revealed no significant differences in EVT’s EMT and cytoskeleton, a notable increase in TGFBR3, increased expression of ECM components LAMA3 and FN1, and a decrease in LGALS1 and MMP2 expression. The most significant finding was the increased expression of EGFR. Analysis of STB showed an increase in TGFB-related genes and EGFR expression, aligning broadly with previous research [67]. This suggests that EGFR can be a significant subject of study and a target in PAS research. To explore the characteristics of trophoblasts at the maternal–fetal interface during placental implantation, we further subdivided the trophoblasts based on previous literature. We divided CTBs into three subgroups: CTB (expressing PAGE4) [47], cytotrophoblast cell columns CTB (CTB-CCC, upregulating ITGB6) [45], and CTB-fusing(upregulating ERVFRD-1 and ERVW-1) [45]. EVTs were divided into four subgroups: EVT1 and EVT2 (upregulating PLAC8, SERPINE1 and SERPINE2) [45], EVT3 (giant cells like, upregulating RAC1) [45, 47], and EVT4 (expressing PRG2) [20]. STBs have two subgroups: STB1 (HLA-G negative, expressing CD81) [68] and STB2(expressing CYP19A1) [45] (Figure S5A and B). The analysis indicates that the PAS tissue samples exhibit an additional EVT subtype, EVT4, not present in the placenta samples. Notably, EVT4 is characterized by high expression of PRG2, aligning with the findings reported in Arutyunyan’s study [20].

### Changes in immune cells and inflammation factors in PAS

**Fig. 6:**
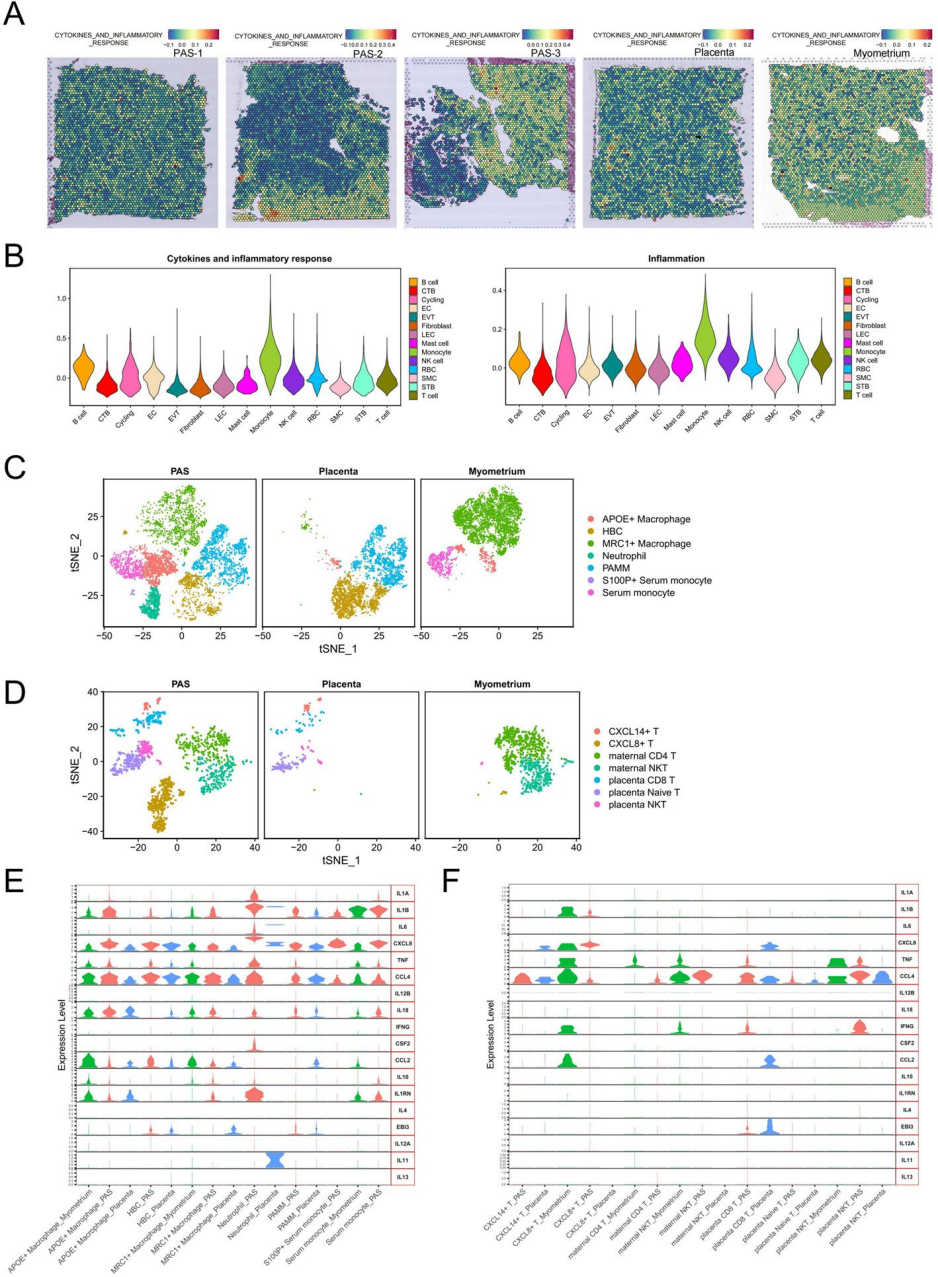
Immune cells and inflammation factors in PAS. **A** Enrichment score of cytokines and inflammatory response pathways of each spot in the ST data. Gradient from blue (low score) to red (high score). **B** Enrichment score of cytokines and inflammatory pathways of each cell type in the scRNA-seq data. **C** t-SNE visualization of monocytic subpopulations divided by PAS, placenta and myometrium groups. **D** t-SNE visualization of T subpopulations divided by PAS, placenta and myometrium groups. **E** Expression of pro-inflammatory and anti-inflammatory factors in monocytic subpopulations divided by PAS, placenta and myometrium groups. **F** Expression of pro-inflammatory and anti-inflammatory factors in T subpopulations divided by PAS, placenta and myometrium groups

**Fig. 7:**
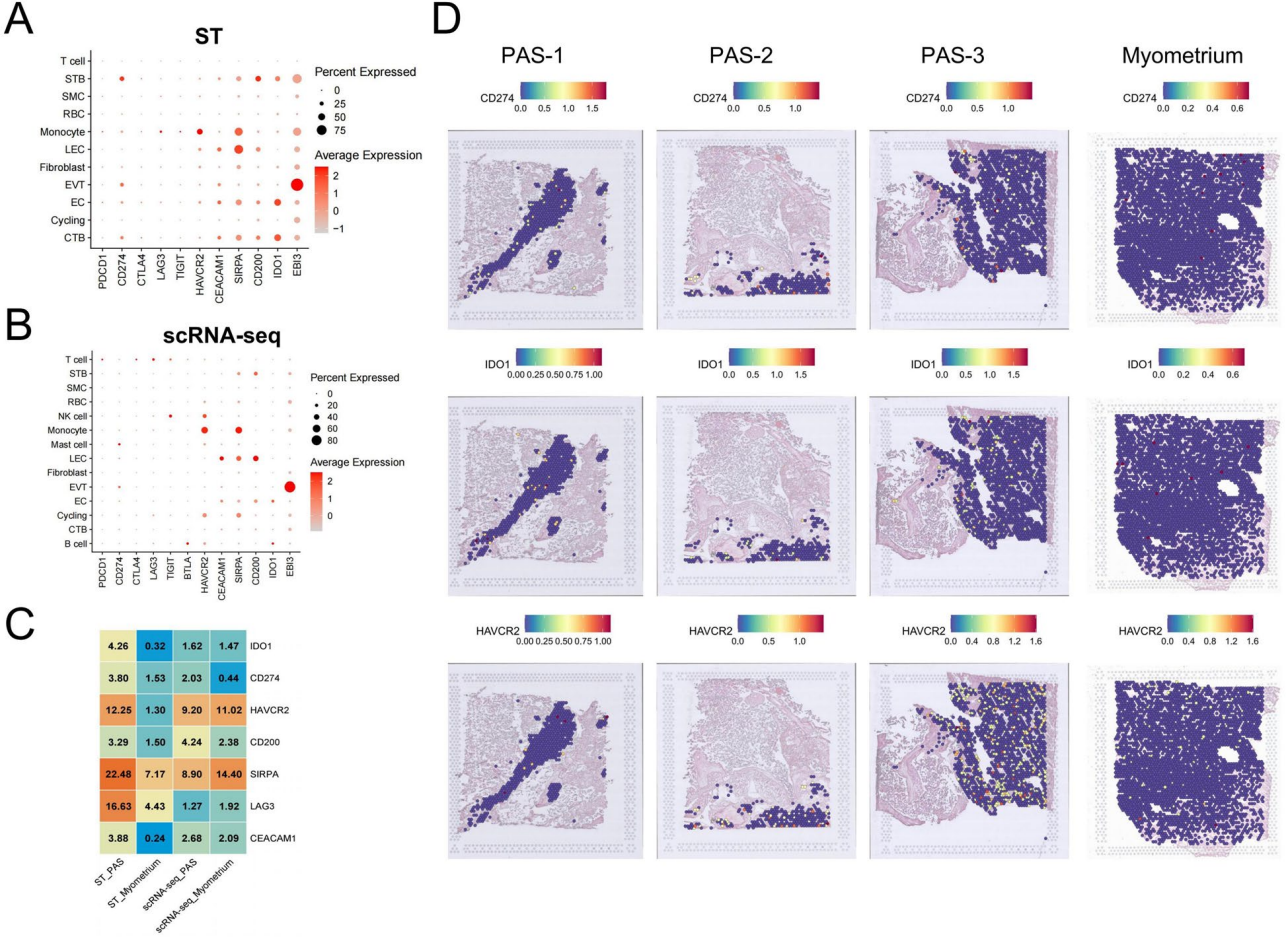
Analysis of immune checkpoints and immunosuppressive genes. in PAS. **A** Dot plot depicting the expression of immune checkpoint genes in the scRNA-seq data. **B** Dot plot depicting the expression of immune checkpoint genes in the ST data. **C** Heatmap showed the fold change of positive spots (SCT-transformed values > 1) and cells proportion in SMC regions and SMC cells compared PAS with myometrium groups. **D** Positive spots of CD274 and IDO1 in the SMC regions in the ST data

**Fig. 8:**
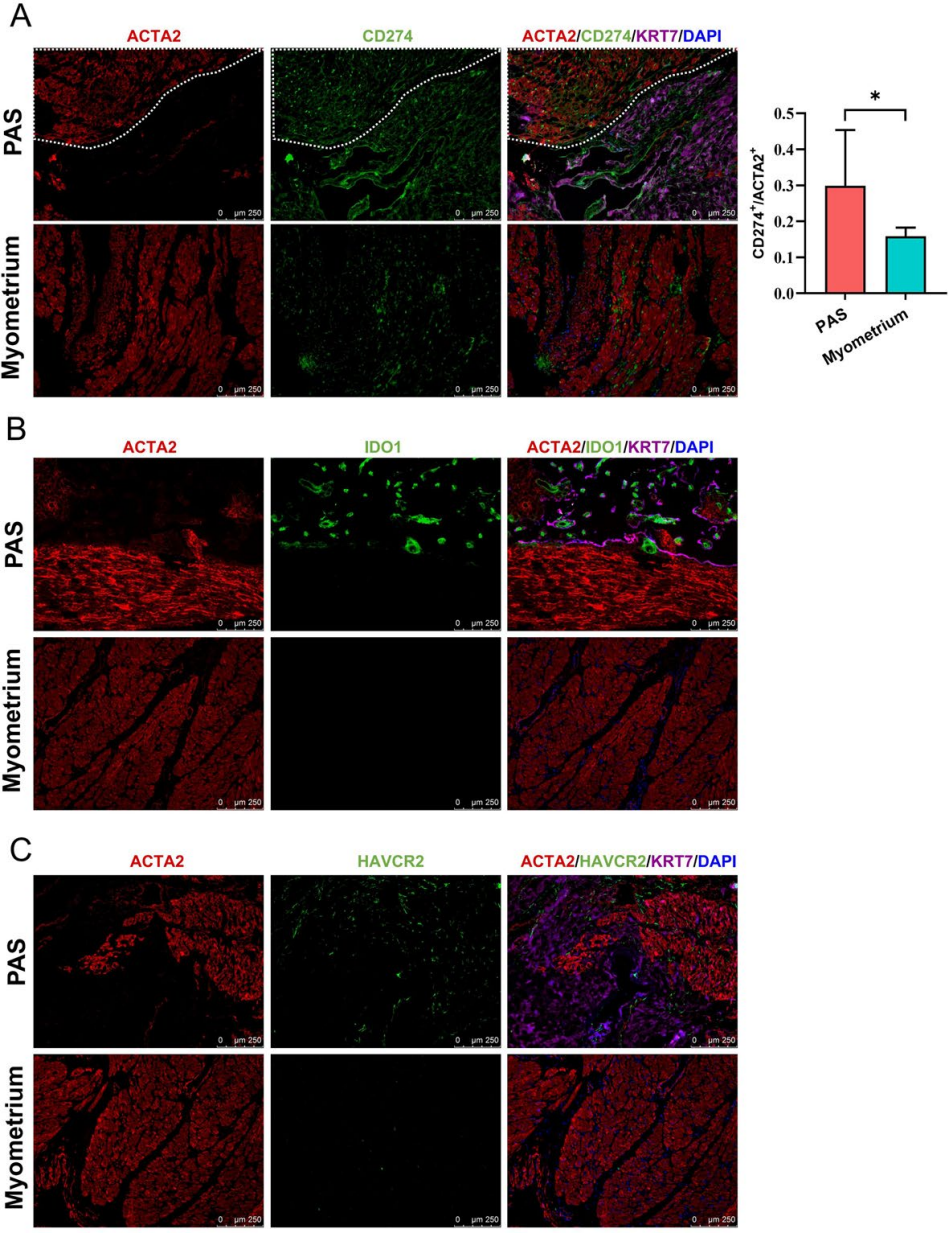
Immunofluorescence staining images indicate the expression of CD274 (**A**), IDO1 (**B**) and HAVCR2 (**C**) in PAS and normal myometrium samples. The area enclosed by the dashed line is the myometrial region of the invasive parts of PAS. The relative fluorescence values of CD274 in the myometrium area of the invasive parts of PAS patients and in normal myometrial tissues were quantified, located to the right in (**A**). Data are represented as mean ± SD. *n* = 7, (*, *p* < 0.05)

Trophoblasts come into direct contact with the myometrium after the decidua loss in the placental accreta region, resulting in immune disorders at the maternal–fetal interface. To gain a better understanding of the changes in immune response at the maternal–fetal interface in PAS, we calculated the cytokines and inflammatory response score and the results showed it seems higher in the SMC region of PAS than other regions (Fig. 6A). Monocytes were the most abundant immune cell types and have the highest score of cytokines and inflammatory response score and inflammation pathways (Fig. 6B). We conducted a subclassification of monocytes, showed neutrophil peculiarly exist in PAS group. APOE + macrophage obviously increased in PAS group (Fig. 6C and S5C). The presence of Hofbauer cells in the invasive areas also exerts an immunosuppressive effect on their microenvironment [69]. In the same way, we found CXCL8 + T peculiarly existed in PAS group (Fig. 6D and S5D). Treg (regulatory T) (FOXP3 +) cells are observed in smaller numbers and do not cluster together (Figure S5E). We futher displayed the expression of pro-inflammatory and anti-inflammatory factors in the subpopulation of monocytes and T cells (Fig. 6E and F). APOE + macrophage and neutrophil in PAS expressed pro-inflammatory factors IL1A, IL1B, IL6, CXCL8 and CCL4. neutrophil expressed anti-inflammatory factors IL1RN. Overall, the levels of pro-inflammatory cytokines secreted by immune cells in PAS are higher than those observed in the placenta. This is particularly evident in APOE + and MRC1 + macrophages, as well as in CXCL14-expressing T cells and NKT cells. In PAS, certain pro-inflammatory factors such as CCL2 and CCL4 are reduced compared to those in the myometrium, notably within APOE + macrophages and CXCL8-expressing T cells. To study the changes of immune tolerance in PAS region, we displayed the expression of genes of canonical immune checkpoint and immunosuppression in the scRNA-seq data (Fig. 7A) and ST data (Fig. 7B). HAVCR2 and SIRPA were mainly expressed by monocytes. EBI3 was observably expressed by EVT. A limited number of cells expressed PD1 (PDCD1), PD-L1 (CD274), CTLA4, LAG3 and BTLA in our data. We counted the positive spots that expressed these immunosuppression genes in the SMC regions of PAS ST data and found increased expressions of IDO1 and CD274 (Fig. 7C and S6A). However, ST data showed that IDO1, CD274 and HAVCR2 expressed more in the SMC regions of PAS compared with normal myometrium, it was apparent that the expression of IDO1, CD274 and HAVCR2 in the myometrium is more sparser (Fig. 7D). Immunofluorescence results revealed a larger high-intensity expression area of the CD274 protein in the invasive parts of PAS compared with the normal myometrium (Fig. 8A). Immunofluorescence of IDO1 showed the protein of IDO1 mainly located at the EC or the immune cells in PAS, but almost not expressed in the myometrium (Fig. 8B), scRNA-seq also displayed the same expression characteristics (Figure S6B). HAVCR2 appears to be mainly expressed in immune cells in PAS group and is almost not expressed in the myometrium (Fig. 8C).

### Expression of immune tolerance factors HLA-G and EBI3 at the PAS interface

**Fig. 9:**
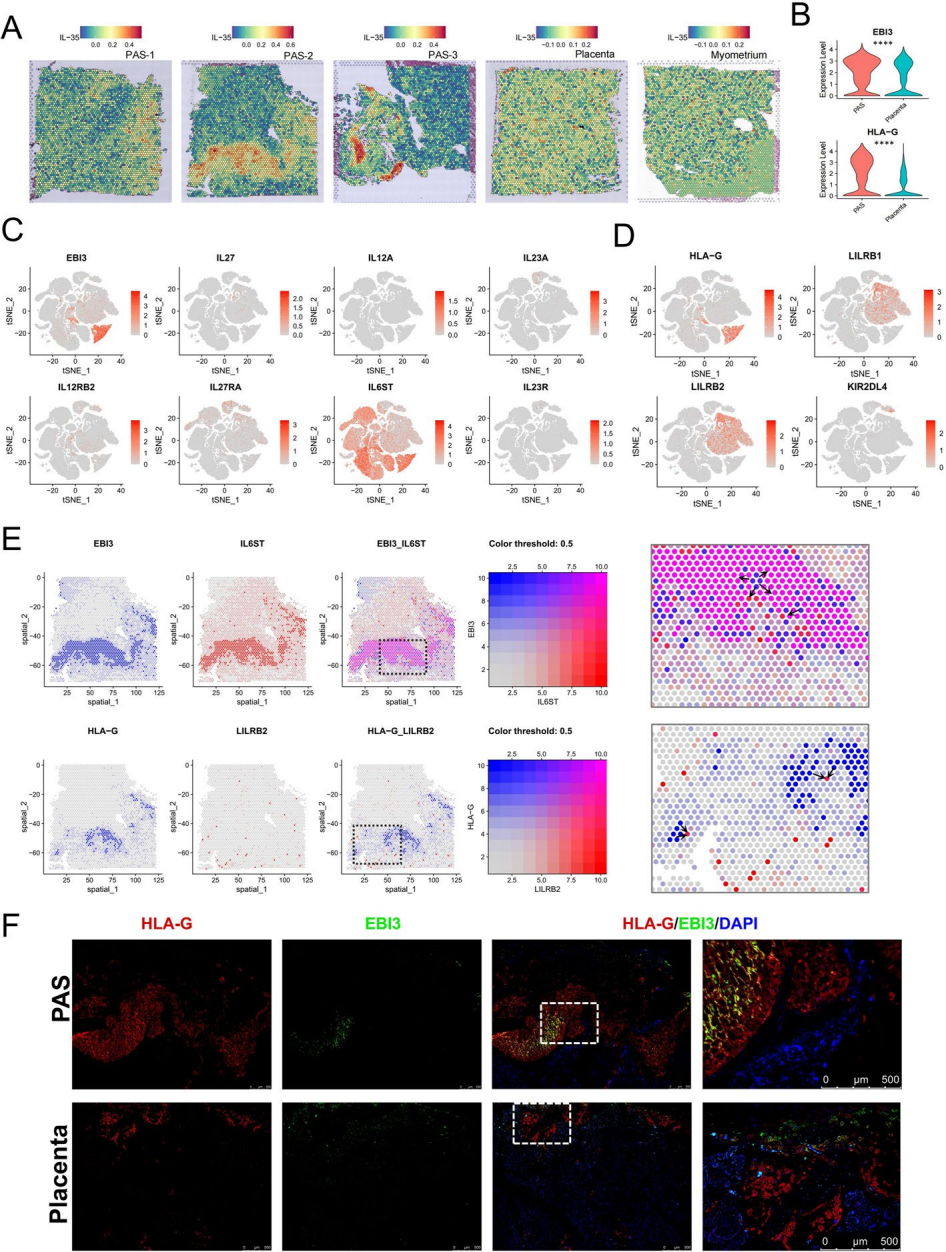
Enhanced IL-35 and HLA-G signal in invasive parts of PAS. **A** Enrichment score of IL-35 response pathways of each spot in the ST data. Gradient from blue (low score) to red (high score). **B** Expression of HLA-G and EBI3 in trophoblast cells in the PAS and normal placenta groups (****, *p* < 0.0001). **C** t-SNE color-coded for the expression of EBI3 signal associated genes in scRNA-seq. **D** t-SNE color-coded for the expression of HLA-G signal associated genes in scRNA-seq. **E** Spatial distribution of HLA-G and EBI3 and their coupled receptors in the ST data of PAS-2. The magnified view of the dashed box area is displayed on the right side of the figure. Arrows indicate the factors and adjacent receptors. **F** Immunofluorescence staining images indicate the expression of HLA-G and EBI3 in the PAS and normal placenta samples. The fourth column shows an enlarged view of the area within the dashed box

HLA-G expression has been observed on the surfaces of preimplantation embryos and EVTs, it plays essential roles in the modulation of spiral artery remodeling, fetal development, and immune tolerance [70, 71]. Although CTBs and STBs generally do not express membrane-bound HLA-G, the expression of HLA-G has been observed in all trophoblast populations, including CTBs and STBs, have the capacity to secrete soluble HLA-G (sHLA-G) [72]. EBI3, also known as IL35B, is expressed by trophoblasts and participates in maternal–fetal tolerance by modulating the ratio of M1/M2 macrophages and suppressing the proliferation of T cells [73, 74]. Here, the IL-35 signal was mainly enriched in the EVT regions (Fig. 9A). HLA-G and EBI3 were highly expressed in EVTs and also showed expression in some other trophoblasts. Compared to the normal placenta group, these two genes were upregulated in trophoblasts of the invasive parts in PAS (Fig. 9B). In the ST data of PAS, HLA-G and EBI3 are co-localized in space (Figure S7A). Further, our scRNA-seq data analysis indicated the expression of all chains and receptors of IL-35 and HLA-G (Fig. 9C and D). We observed significant expression of EBI3 in EVTs and certain trophoblasts, whereas the expression of IL12A (P35) was limited to only few cells. Among the receptors analyzed, IL6ST (GP130) was predominantly expressed in stromal cells and trophoblasts. However, the expression levels of other receptors were generally low in all cells. Additionally, HLA-G was expressed by EVTs and some trophoblasts. Its receptors, LILRB1 and LILRB2, were found to be expressed by monocytes, whereas KIR2DL4 was expressed by NK cells. We identified the spatial co-localization of EBI3/IL6ST and HLA-G/LILRB2 in one of our ST data in the PAS group, indicating their spatial proximity (Fig. 9E). EBI3 and HLA-G were primarily expressed in the EVT region. The EBI3 signals released by EVT cells were mainly received by EVT cells themselves, as well as nearby SMCs and other trophoblasts. As one of the receptors for HLA-G, LILRB2 was mainly expressed in monocytes within PAS tissues. The spatial proximity allows for more effective transmission of immunosuppression signals. We performed immunofluorescence to display the expression and co-location of EBI3 and HLA-G in the invasive parts of PAS and normal placenta (Fig. 9F).

## Discussion

We used high-resolution ST detection techniques to investigate the microenvironment and molecular mechanisms underlying PAS. The analysis revealed that immune dysregulation as the hallmark of PAS. The immune suppression pathways, including EBI3 and HLA-G signaling pathways, were upregulated in the trophoblasts of PAS. The expression of immune checkpoint genes such as CD274 and IDO1, was increased in PAS. Collectively, trophoblasts and SMCs contribute to establishing an immunosuppressive microenvironment in the invasive parts. We found that PTK2 and EGFR are up-regulated in EVT and STB in PAS, which can promote angiogenesis and cell proliferation..

HLA-G and EBI3 signaling was enhanced in the trophoblasts at the invasive parts. HLA-G plays pivotal roles in the remodeling of spiral arteries, fetal development, and immune tolerance [71]. These functions are facilitated by the expression of HLA-G molecules on EVTs, which interact with leukocytes expressing specific receptors, KIR2DL4, LILRB1 (ILT2) and LILRB2 (ILT4), creating an immune-tolerant environment at the maternal–fetal interface [75, 76]. Notably, NK cells have been reported to express two HLA-G receptors, namely KIR2DL4 and LILRB1 [77]. This interaction between HLA-G and NK cells helps regulate NK cell activity and promote immune tolerance during pregnancy [71, 78]. CD14 + monocytes express HLA-G receptors LILRB1 and LILRB2 [79, 80]. These receptors enable the interaction between HLA-G and monocytes, leading to an increase in the suppressive effect of placental monocytes on T-cell proliferation [79].

EBI3 (IL-35), which is an anti-inflammatory cytokine of the IL-12 family and plays an important role in immune suppression, was found increased in EVTs from patients with PAS [81, 82]. There is a higher expression of EBI3 (IL-35) in EVT cells from term patients with PAS, while a lower expression in normal term pregnancy. These results suggest the potential role of EBI3 in maintaining the immune balance of the placental–uterine interface in placental accretion, in addition to controlling the depth of invasion in embryo implantation.

Immune checkpoint molecules, such as PD-L1 (CD274), play a critical role in maintaining immune tolerance at the maternal–fetal interface [83]. Our study revealed an upregulation of PD-L1 expression in the SMC regions of invasive tissues in PAS. This upregulation may prevent excessive immune reactions against the myometrium in PAS cases. Additionally, other immune checkpoint molecules, including IDO1 and HAVCR2 (TIM-3), were markedly increased. Reduced expression of IDO1 at the maternal–fetal interface has been associated with conditions, such as intrauterine growth restriction, preeclampsia, and recurrent miscarriage. HAVCR2 has also been implicated in regulating immune responses during pregnancy [84]. These findings highlight the importance of immune tolerance at the myometrium in PAS-affected pregnant women.

Our findings contribute to the understanding of PAS pathophysiology and indicate the need to further explore the immunologic aspect, particularly trophoblast invasiveness, in this context. The identification of key genes and pathways associated with PAS, such as HLA-G, EBI3, PTK2 and ERBB pathway, provides a theoretical basis for the development of targeted drugs and therapeutic interventions. Our findings indicate the potential application of immune modulatory drugs in PAS. However, they also raise considerations among researchers regarding the impact of taking immunosuppressive drugs during pregnancy on the occurrence of PAS.

Our study has several limitations. The availability of samples was limited because of the rarity of PAS. Some parts of the PAS region at the maternal–fetal interface may be retained in the uterus during surgical procedures aimed at hemostasis and preserving the uterus, resulting in a missing structure of the accreta site [6, 85]. Our samples were obtained exclusively from cases with a confirmed diagnosis of PAS. These cases showed severe placental invasion, leading to significant loss of decidual tissue. Therefore, our scRNA-seq and ST data had a limited number of decidual cells and spots. Moreover, PAS may have atypical manifestations in pathologic sections, further complicating the analysis. The ST capture area used in our study measured 6.5 × 6.5 mm with 4992 spots per capture area. Each spot has a diameter of 55 µm, allowing coverage of 1–5 cells. However, cells with lower abundance and dispersed distribution are more likely to be influenced by the gene expression of surrounding cells, making them less detectable. The cell type that registers the highest score at each spot is designated as the cell type for that spot. Therefore, certain cell types, such as B cells and NK cells, were not detected in the ST analysis. We are also exploring and experimenting with higher-resolution ST technologies. Additionally, our PAS cases caused by myometrial or decidual injuries. This kind of PAS, resulting from placenta itself or immune deficiencies, is extremely rare and obtaining relevant tissues is quite challenging. In the future, we will pay more attention to these types of cases. Furthermore, our current findings need to be validated through comprehensive animal, cellular and molecular experiments to provide a more in-depth understanding of the mechanisms involved.

## Conclusion

We used high-resolution molecular detection techniques, ST, and scRNA-seq to elucidate the mechanisms and microenvironment involved in the invasive parts of PAS. Invasive trophoblasts show an increased expression of HLA-G and EBI3 (IL-35), suggesting their crucial role in establishing an immunosuppressive microenvironment within the myometrium. This immune modulation is further supported by the elevated expression of immune checkpoint genes, including CD274, IDO1, and HAVCR2. The enhanced immunoevasive properties of trophoblasts facilitate their escape from immune surveillance. Moreover, a highly immunosuppressive microenvironment is possibly established in the invaded myometrium, which could further promote trophoblast survival. These findings highlight the complex interplay between trophoblasts and the maternal immune system in PAS, possibly underscoring the importance of immune tolerance mechanisms in the invasive process. High expression of PTK2 and EGFR in trophoblasts promotes angiogenesis and the proliferation of trophoblast cells. These findings could potentially serve as early biological markers, offering new clinical diagnostic indicators for PAS. Additionally, our research provides new data support for exploring the pathogenic mechanisms of PAS, potentially presenting new intervention targets for its treatment.

### Supplementary Information

Supplementary Material 1: Supplemental Figure 1. (A) Ultrasound and MRI of PAS patients.

Supplementary Material 2: Supplemental Figure 2. (A) H&E staining of tissues in invasive PAS basal plate, normal placenta and myometrium [35]. (B) t-SNE color-coded for the expression of canonical markers of each cell population in scRNA-seq. (C) Immunofluorescence indicates the expression of the marker of decidua (WNT4), SMC (ACTA2), and trophoblast (KRT7) in the invasive part of PAS and the normal myometrium attached with decidua.

Supplementary Material 3: Supplemental Figure 3. (A) Spatial feature plots of each cell type prediction in invasive parts of PAS, placenta and myometrium groups in the ST data. Gradient from blue (low score) to red (high score).

Supplementary Material 4: Supplemental Figure 4. (A) t-SNE plot of the cycling population in the scRNA-seq data segregated by groups. (B) Information flow of each signaling pathway in the PAS group compared with the normal placenta or normal myometrium groups. Red indicates significantly (*p* < 0.05) increased signaling in the PAS group compared with the placenta or myometrium groups, blue indicates the significantly (*p* < 0.05) opposite trend, and black indicates no significantly difference (*p* > 0.05). (C) In the comparison between PAS and myometrium, the dot plot depicts the expression of significantly upregulated (*p* < 0.05) cell communication among three trophoblast subtypes with other cell types.

Supplementary Material 5: Supplemental Figure 5. (A) t-SNE plot of the subpopulation of trophoblast in the scRNA-seq data segregated by groups. (B) Dot plot depicts the expression of canonical markers for trophoblast in the scRNA-seq data. The color of each dot indicates the expression level of the marker gene, and the size of the dot reflects the percentage of cells expressing the marker genes across various populations. (C) Violin plot depicts the expression of the markers of monocytic subpopulations. (D) Violin plot depicts the expression of the markers of T cell subpopulations. (E) t-SNE color-coded for the expression of canonical markers of Treg cells in T cell subpopulations.

Supplementary Material 6: Supplemental Figure 6. (A) Spatial distribution of the canonical immune checkpoint and immunosuppression genes in the ST data. (B) t-SNE color-coded for the expression of canonical immune checkpoint and immunosuppression genes in scRNA-seq split by groups.

Supplementary Material 7: Supplemental Figure 7. (A) Spatial distribution of HLA-G and EBI3 in the ST data of PAS group.

Supplementary Material 8.

Supplementary Material 9.

## Data Availability

scRNA-seq and ST data in this study are available in the GSA database with accession numbers HRA005072.

## Abbreviations

PAS: Placenta Accreta Spectrum
scRNA-seq: Single-cell RNA sequence
ST: Spatial transcriptomics
EVT: Extravillous trophoblast
CTB: Cytotrophoblast
STB: Syncytiotrophoblast
t-SNE: T-distributed stochastic neighbor embedding
SMC: Smooth muscle cell
EC: Endothelial cell
LEC: Lymphatic endothelial cell
ECM: Extracellular matrix
DEGs: Differential expressed genes
RT-qPCR: Real Time Quantitative PCR
UMI: Unique molecular identified
