# Supplementary figures and images for: Single-cell and spatial transcriptomics reveal alterations in trophoblasts at invasion sites and disturbed myometrial immune microenvironment in placenta accreta spectrum disorders
Source: Biomark Res. 2024 Jun 3;12:55. doi: 10.1186/s40364-024-00598-6 (PMC11149369; doi:10.1186/s40364-024-00598-6)

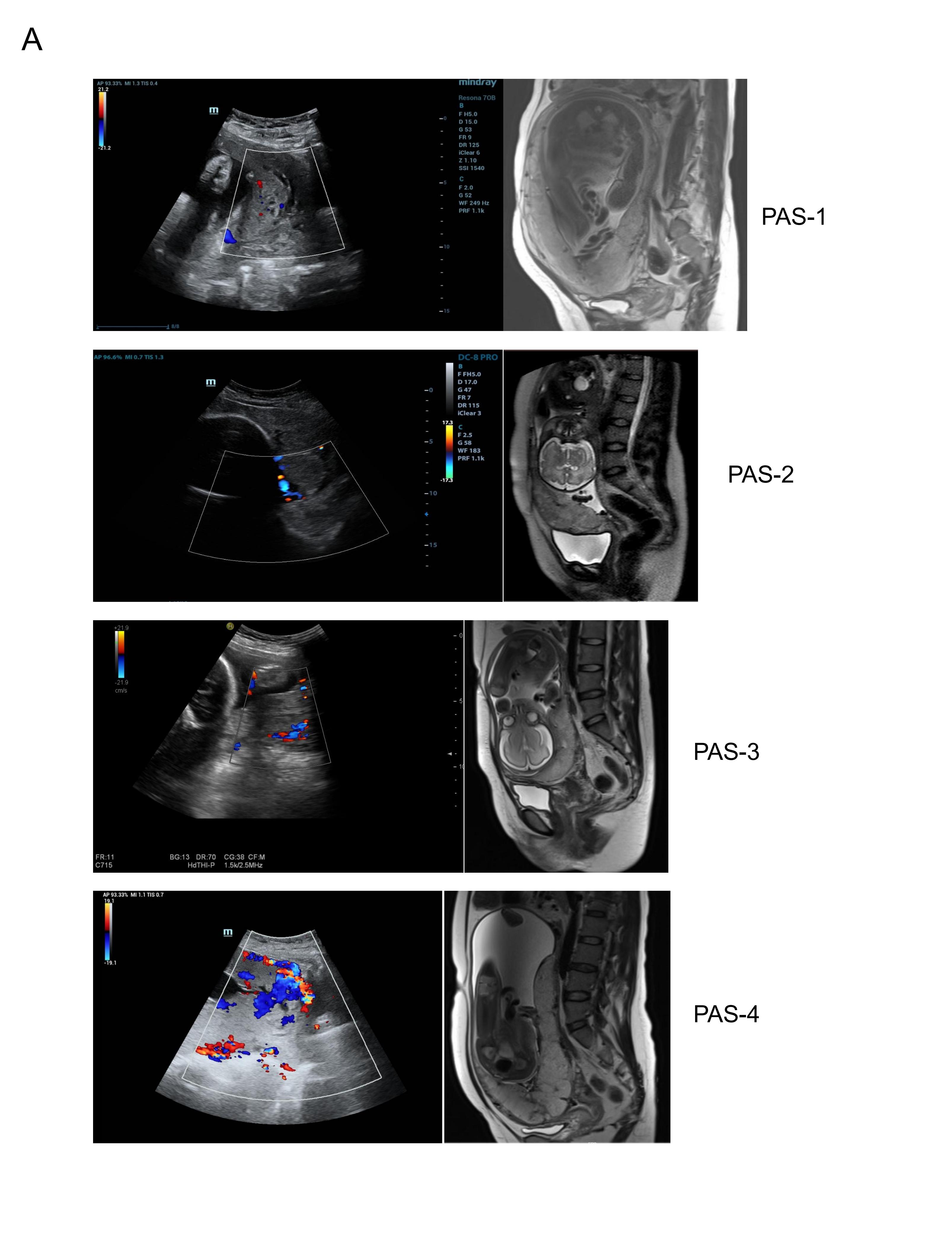

Supplement: Supplementary file 1 — Supplementary Material 1: Supplemental Figure 1. (A) Ultrasound and MRI of PAS patients. [file 40364_2024_598_MOESM1_ESM.jpg]

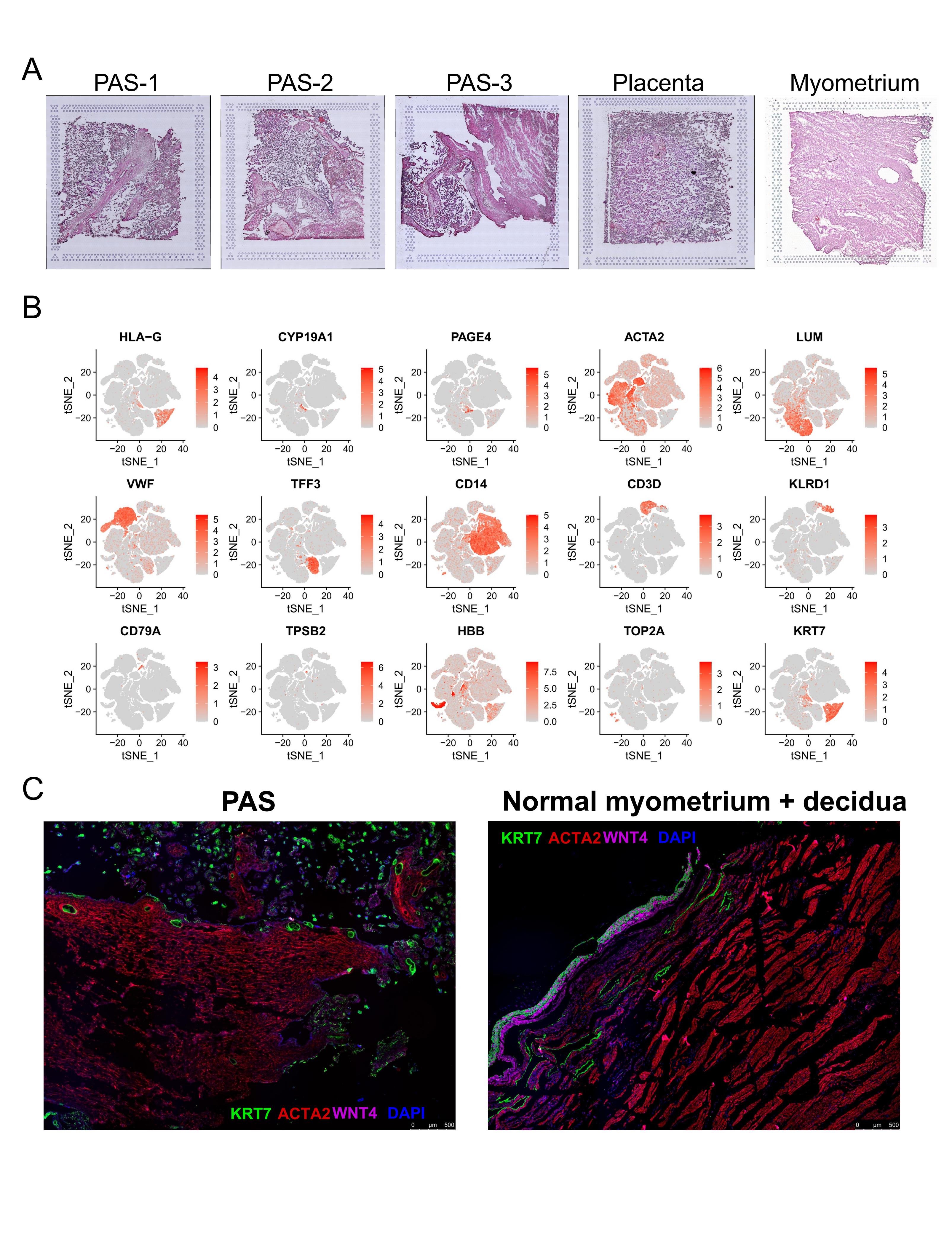

Supplement: Supplementary file 2 — Supplementary Material 2: Supplemental Figure 2. (A) H&E staining of tissues in invasive PAS basal plate, normal placenta and myometrium [35]. (B) t-SNE color-coded for the expression of canonical markers of each cell population in scRNA-seq. (C) Immunofluorescence indicates the expression of the marker of decidua (WNT4), SMC (ACTA2), and trophoblast (KRT7) in the invasive part of PAS and the normal myometrium attached with decidua. [file 40364_2024_598_MOESM2_ESM.jpg]

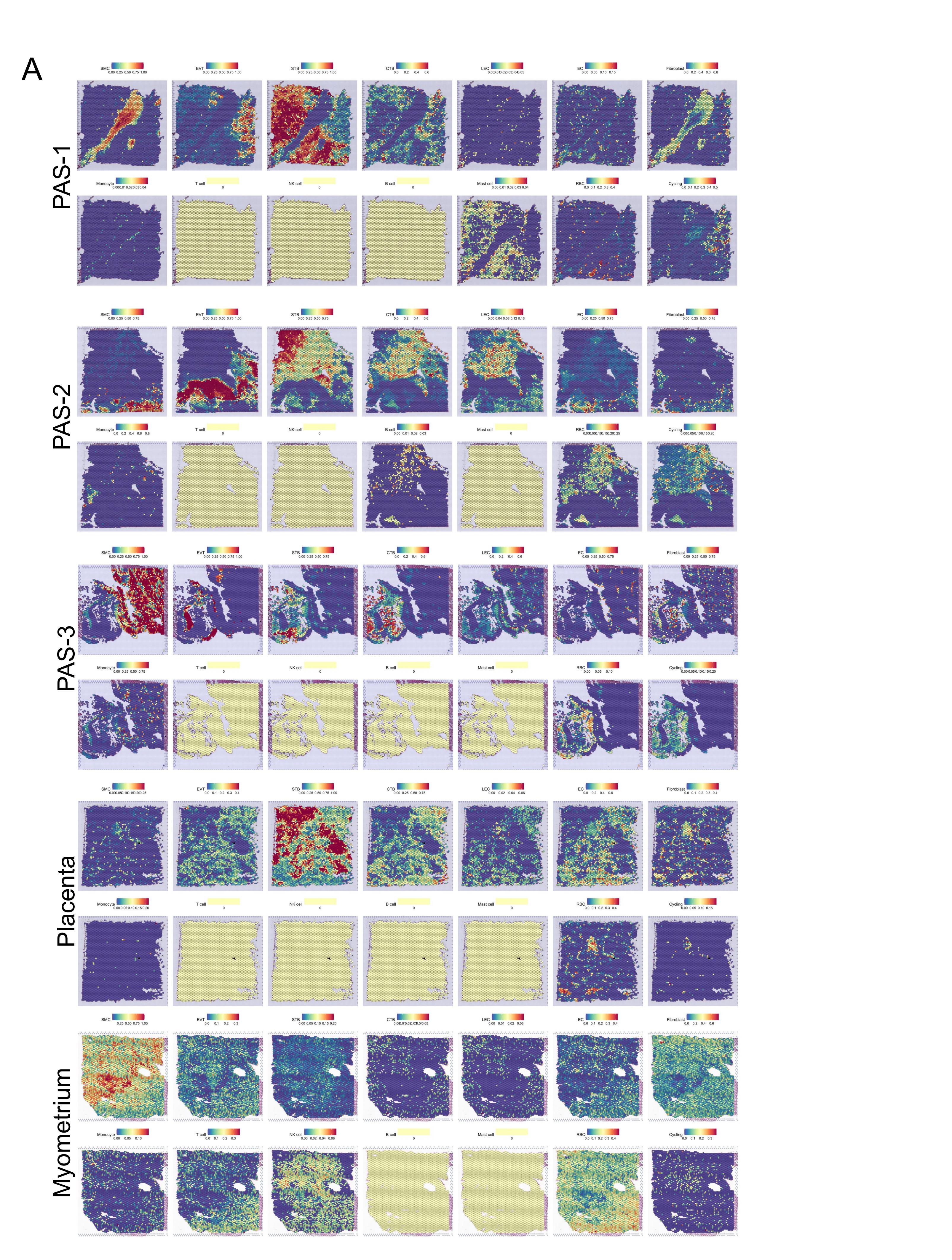

Supplement: Supplementary file 3 — Supplementary Material 3: Supplemental Figure 3. (A) Spatial feature plots of each cell type prediction in invasive parts of PAS, placenta and myometrium groups in the ST data. Gradient from blue (low score) to red (high score). [file 40364_2024_598_MOESM3_ESM.jpg]

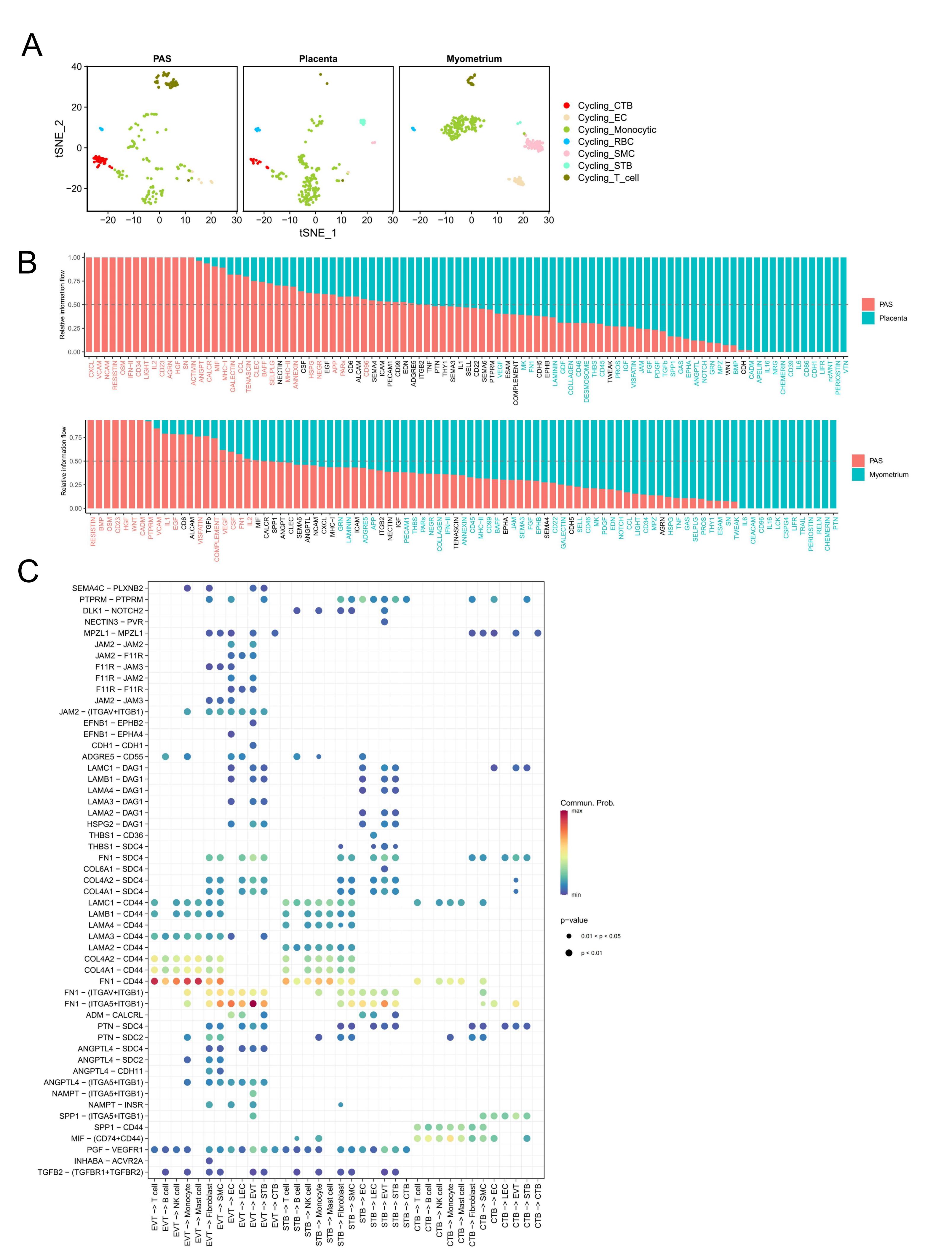

Supplement: Supplementary file 4 — Supplementary Material 4: Supplemental Figure 4. (A) t-SNE plot of the cycling population in the scRNA-seq data segregated by groups. (B) Information flow of each signaling pathway in the PAS group compared with the normal placenta or normal myometrium groups. Red indicates significantly (p < 0.05) increased signaling in the PAS group compared with the placenta or myometrium groups, blue indicates the significantly (p < 0.05) opposite trend, and black indicates no significantly difference (p > 0.05). (C) In the comparison between PAS and myometrium, the dot plot depicts the expression of significantly upregulated (p < 0.05) cell communication among three trophoblast subtypes with other cell types. [file 40364_2024_598_MOESM4_ESM.jpg]

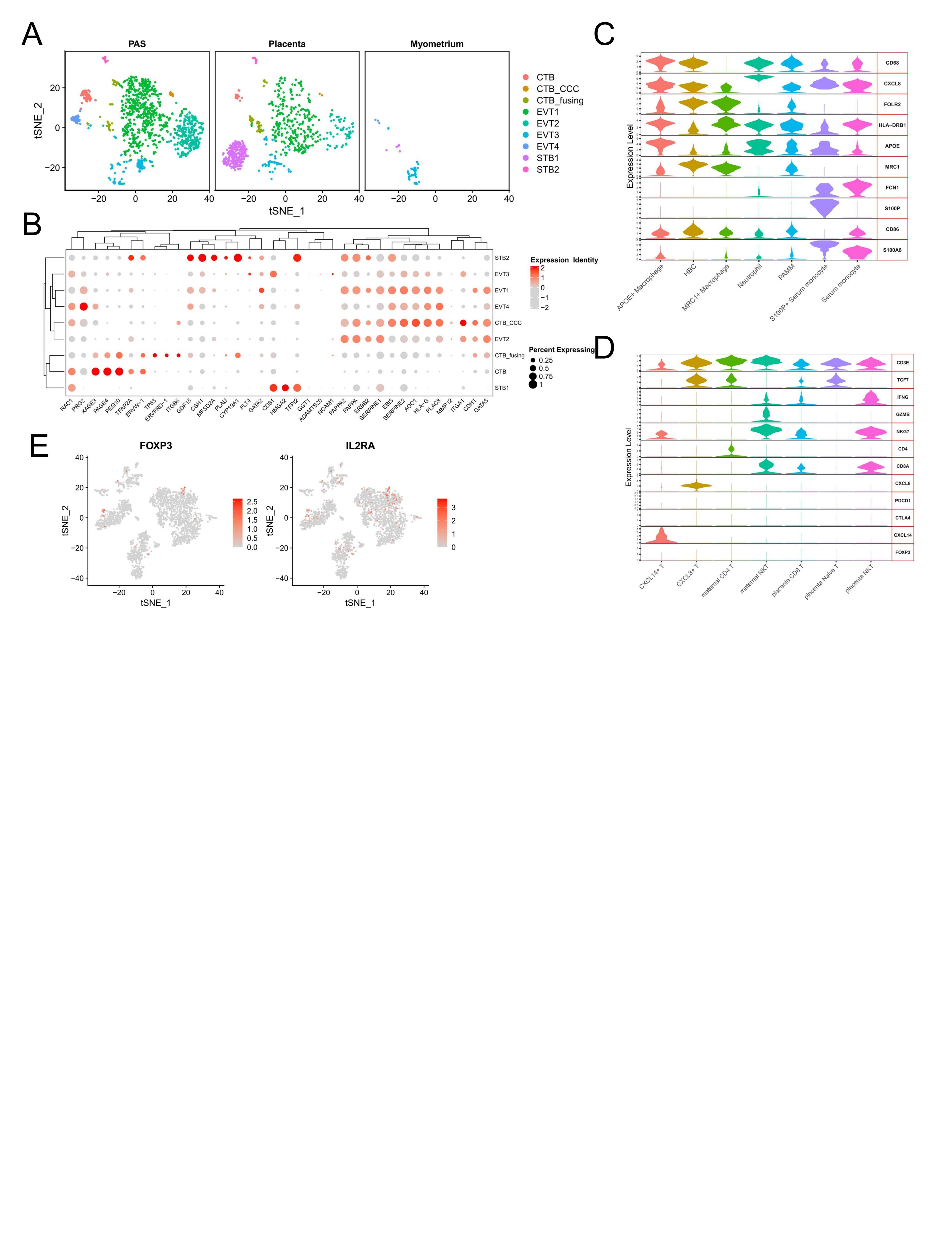

Supplement: Supplementary file 5 — Supplementary Material 5: Supplemental Figure 5. (A) t-SNE plot of the subpopulation of trophoblast in the scRNA-seq data segregated by groups. (B) Dot plot depicts the expression of canonical markers for trophoblast in the scRNA-seq data. The color of each dot indicates the expression level of the marker gene, and the size of the dot reflects the percentage of cells expressing the marker genes across various populations. (C) Violin plot depicts the expression of the markers of monocytic subpopulations. (D) Violin plot depicts the expression of the markers of T cell subpopulations. (E) t-SNE color-coded for the expression of canonical markers of Treg cells in T cell subpopulations. [file 40364_2024_598_MOESM5_ESM.jpg]

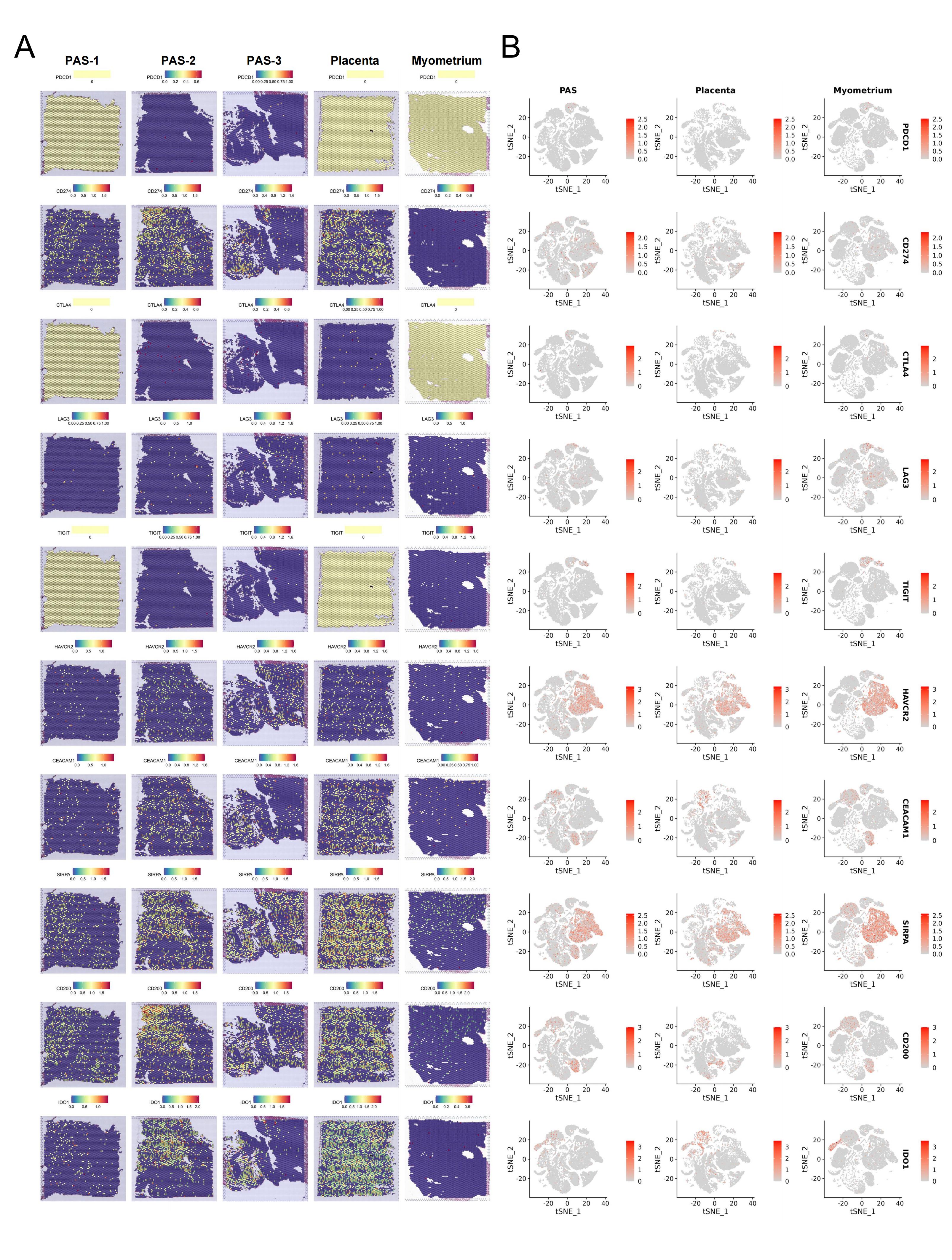

Supplement: Supplementary file 6 — Supplementary Material 6: Supplemental Figure 6. (A) Spatial distribution of the canonical immune checkpoint and immunosuppression genes in the ST data. (B) t-SNE color-coded for the expression of canonical immune checkpoint and immunosuppression genes in scRNA-seq split by groups. [file 40364_2024_598_MOESM6_ESM.jpg]

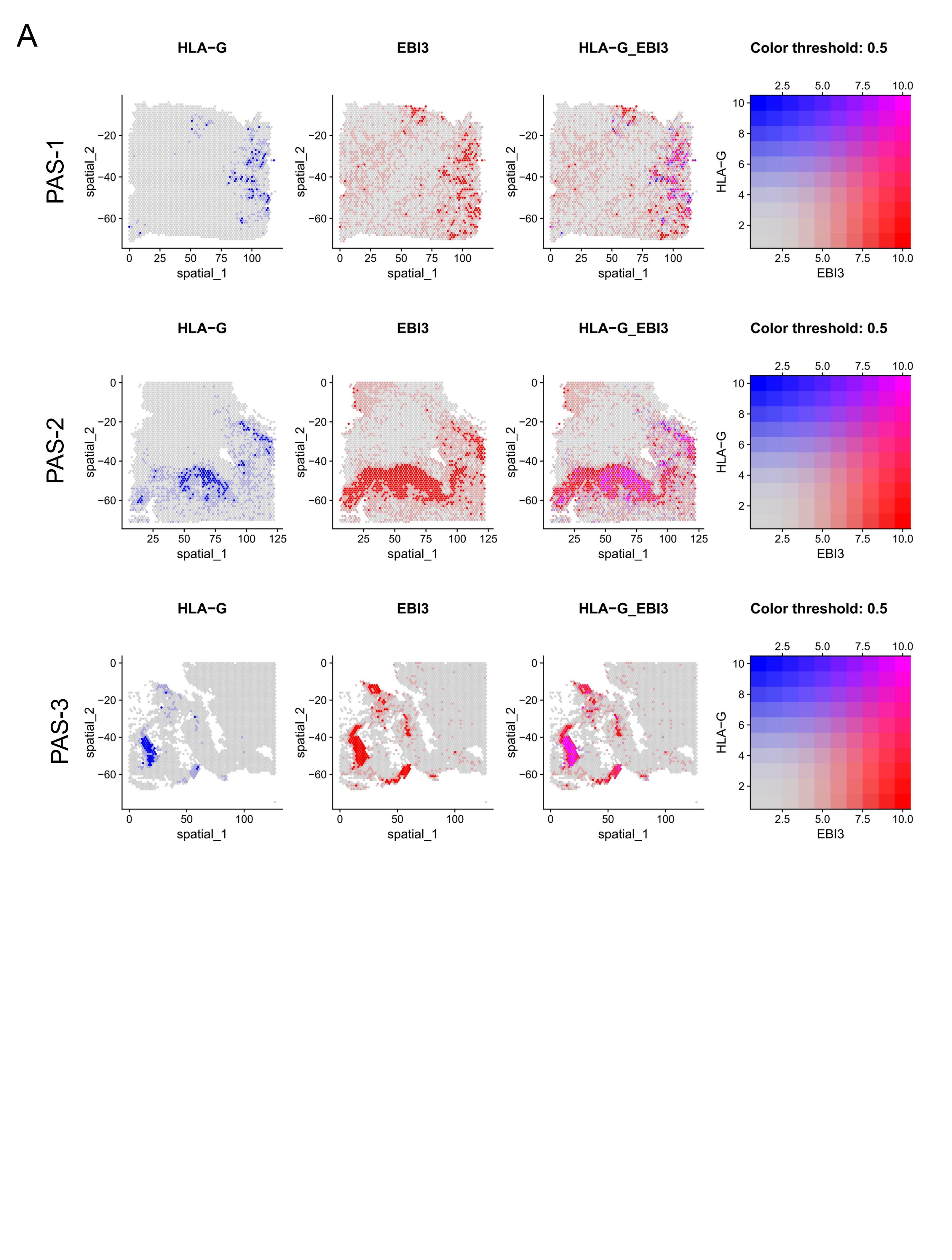

Supplement: Supplementary file 7 — Supplementary Material 7: Supplemental Figure 7. (A) Spatial distribution of HLA-G and EBI3 in the ST data of PAS group. [file 40364_2024_598_MOESM7_ESM.jpg]
